# Pre-Symptomatic Activation of Antioxidant Responses and Alterations in Glucose and Pyruvate Metabolism in Niemann-Pick Type C1-Deficient Murine Brain

**DOI:** 10.1371/journal.pone.0082685

**Published:** 2013-12-18

**Authors:** Barry E. Kennedy, Veronique G. LeBlanc, Tiffany M. Mailman, Debra Fice, Ian Burton, Tobias K. Karakach, Barbara Karten

**Affiliations:** 1 Department of Biochemistry and Molecular Biology, Dalhousie University, Halifax, Nova Scotia, Canada; 2 Institute of Marine Biosciences, National Research Council of Canada, Halifax, Nova Scotia, Canada; The University of New South Wales, Australia

## Abstract

Niemann-Pick Type C (NPC) disease is an autosomal recessive neurodegenerative disorder caused in most cases by mutations in the *NPC1* gene. NPC1-deficiency is characterized by late endosomal accumulation of cholesterol, impaired cholesterol homeostasis, and a broad range of other cellular abnormalities. Although neuronal abnormalities and glial activation are observed in nearly all areas of the brain, the most severe consequence of NPC1-deficiency is a near complete loss of Purkinje neurons in the cerebellum. The link between cholesterol trafficking and NPC pathogenesis is not yet clear; however, increased oxidative stress in symptomatic NPC disease, increases in mitochondrial cholesterol, and alterations in autophagy/mitophagy suggest that mitochondria play a role in NPC disease pathology. Alterations in mitochondrial function affect energy and neurotransmitter metabolism, and are particularly harmful to the central nervous system. To investigate early metabolic alterations that could affect NPC disease progression, we performed metabolomics analyses of different brain regions from age-matched wildtype and *Npc1* ^-/-^ mice at pre-symptomatic, early symptomatic and late stage disease by ^1^H-NMR spectroscopy. Metabolic profiling revealed markedly increased lactate and decreased acetate/acetyl-CoA levels in *Npc1* ^-/-^ cerebellum and cerebral cortex at all ages. Protein and gene expression analyses indicated a pre-symptomatic deficiency in the oxidative decarboxylation of pyruvate to acetyl-CoA, and an upregulation of glycolytic gene expression at the early symptomatic stage. We also observed a pre-symptomatic increase in several indicators of oxidative stress and antioxidant response systems in *Npc1* ^-/-^ cerebellum. Our findings suggest that energy metabolism and oxidative stress may present additional therapeutic targets in NPC disease, especially if intervention can be started at an early stage of the disease.

## Introduction

Niemann-Pick Type C (NPC) disease is a fatal, autosomal recessive neurodegenerative disease caused in 95% of cases by mutations in the *NPC1* gene and in the remaining cases by mutations in *NPC2*. NPC1 is a late endo/lysosomal protein that interacts with NPC2 in the lysosome lumen to mediate cholesterol egress from endosomes to plasma membrane and endoplasmic reticulum [1]–[3]. The loss of either NPC1 or NPC2 leads to the endo/lysosomal accumulation of unesterified cholesterol, an impaired cholesterol homeostatic response and a wide range of other cellular abnormalities, such as decreased oxysterol production, alterations in calcium homeostasis and autophagy, increased oxidative stress and inflammatory responses [4]–[9]. The most severe consequences of NPC1-deficiency are a progressive loss of Purkinje neurons in the cerebellum and widespread neuronal abnormalities. Other common characteristics of NPC1-deficient brain include altered synaptic transmission, glial activation, neuroinflammation, and dysmyelination [10]–[13]. In the periphery, increased total cholesterol levels cause hepatosplenomegaly and liver dysfunction [14]. Neuronal cholesterol accumulation begins at early, pre-symptomatic stages of the disease, and mobilization of endosomal cholesterol *in vitro* or *in vivo* can prevent many of the cellular abnormalities and symptoms of NPC disease [15]–[19]. Although the defect in cholesterol trafficking causes many, if not all, cellular abnormalities observed in NPC disease, relatively little is known about the cellular mechanisms leading to neuronal dysfunction and neurodegeneration.

Endosomal cholesterol is transported to mitochondria even in the absence of functional NPC1, and the endosomal cholesterol accumulation can lead to increased cholesterol levels in mitochondria [20], [21]. Increased levels of mitochondrial cholesterol may lead to lower ATP production and a greater sensitivity of these mitochondria to oxidative stress [22], [23]. In human embryonic stem cell-derived NPC1-deficient neurons, defective mitophagy led to accumulation of depolarized, fragmented mitochondria [24]. Several reports of increased oxidative stress in NPC1-deficiency further implicate mitochondria in NPC disease pathology. For instance, NPC patients have increased serum levels of oxidative stress markers [8], [25], [26], and oxidative tissue damage was observed in the liver and cerebellum of late symptomatic NPC1-deficient mice together with a gene expression profile indicative of increased oxidative stress [9], [27].

The brain is particularly sensitive to alterations in mitochondrial integrity or function due to its high energy requirement and reliance on oxidative metabolism [28]. Most neurodegenerative diseases are associated with mitochondrial dysfunction and primary mitochondrial defects commonly manifest in the brain [29]. The compartmentation of pathways between neurons and astrocytes and the close connection between energy metabolism and neurotransmitter balance further increase the complexity of brain metabolism [30]–[32].

Here we have used an unbiased metabolomics approach to investigate changes in brain energy metabolism beginning at pre-symptomatic stages of NPC disease. Metabolites were measured by ^1^H-NMR spectroscopy in the cerebella, cerebral cortices and hippocampi of BALB/cNctr-Npc1^m1N^/J mice, a well-characterized murine model of NPC disease with a null mutation in *Npc1*. Homozygous *Npc1* ^-/-^ mice are asymptomatic at birth, but develop progressive ataxia, tremors and hindlimb dystonia around 5 – 6 weeks of age, and have an average lifespan of approximately 12 weeks of age [33]. The pathological changes in the brain of these mice at different stages of the disease have been described in some detail [10], [33]–[36]. Protein and mRNA expression analyses of key metabolic proteins gave further insight into mitochondrial changes and into the roles of neurons and astrocytes in NPC1-deficient cerebellum. Our results revealed significant pre-symptomatic alterations in glucose and pyruvate metabolism, as well as an activation of stress-related pathways in NPC1-deficient brain. Further investigations will be required to determine whether the identified pathways could present additional targets for therapeutic intervention to alleviate the symptoms of NPC disease.

## Materials and Methods

### Materials

Neurobasal medium, Minimum essential medium (MEM), B27 and fetal bovine serum were purchased from Life Technologies (Burlington, ON). Poly-D-lysine was obtained from Peptides International (Louisville, KY). [^3^H]-deoxyglucose (2-[1,2-^3^H(N)]; 1 mCi/ml; 25–50 Ci/mmol) was obtained from Perkin-Elmer (Waltham, MA). Other chemicals and buffers were obtained from Sigma (Oakville, ON) or Thermo Fisher Scientific (Ottawa, ON).

### Animals

NPC1-deficient mice were derived from an in-house breeding colony of BALB/cNctr-Npc1^m1N^/J mice heterozygous for a null mutation in *Npc1* (originally obtained from The Jackson Laboratory, Bar Harbour, ME, strain 003092). Only wildtype mice (WT) and mice homozygous for the mutation (*Npc1* ^-/-^) were used in this study. All animals were genotyped by PCR with primers as described [34]. Mice were fed a normal chow diet (Prolab RMH 3000 5P00*, LabDiet) ad libitum and not fasted prior to termination. To collect brain tissue for analysis by ^1^H-NMR or quantitative PCR (qPCR), animals were deeply anesthetized with halothane (Sigma, Oakville, ON) and terminated by decapitation at postnatal day P21/22, P35/36, or P49/50. Brain tissue was removed rapidly, and the cerebellum, cerebral cortices and hippocampi were dissected and immediately snap-frozen in liquid nitrogen.

### Ethics statement

All procedures were approved by the animal ethics committee of Dalhousie University (protocol numbers #10-102 and #10-030) based on the standards established by the Canadian Council of Animal Care.

### ^1^H-NMR spectroscopy

Metabolites were extracted for ^1^H-NMR spectroscopy essentially as described [37]. Briefly, tissues were mixed with methanol to a final methanol/water ratio of 3.2∶1 and homogenized via ultra-sonication in a FastPrep®-24 instrument (MP Biomedicals Inc., Solon, OH). Homogenates were extracted with chloroform and water in a final chloroform/methanol/water ratio of 1∶1∶0.81. Aqueous extracts were dried under nitrogen, re-dissolved in 700 µL of phosphate buffer (1 mM NaH_2_PO_4_, pH 7.2) with 172.2 mg/mL of sodium 3-trimethylsilyl-2,2,3,3-d_4_-propionate (TMSP, Cambridge Isotope Laboratories, Andover, MA) in D_2_O as internal reference standard, and stored in liquid nitrogen until analysis. 1D-^1^H-NMR spectra were acquired using a 5 mm TCI CryoProbe™ (Bruker Biospin) on a Bruker Avance III spectrometer (Bruker Biospin) operating at 700 MHz proton resonance frequency [38]. A detailed description of the acquisition parameters and conditions is attached as Text S1.

### Gene expression analysis by quantitative PCR

Total RNA was prepared from snap-frozen brain tissue using a commercially available kit (Aurum Total RNA kit, BioRad, Mississauga, ON). One µg of RNA was used to prepare cDNA using iScript reverse transcriptase (BioRad) according to the manufacturer's instructions. Quantitative PCR (qPCR) was performed on 0.66 ng of reverse transcribed RNA using the iSYBR Green Mastermix (BioRad) and primers as listed in Table S1. Specificity of the amplification reaction was tested by melt curve analysis, agarose gel electrophoresis, and sequencing of the amplicon. Data were calculated by the Pfaffl method with cyclophilin (*Ppia*) as a housekeeping gene and standardized to the wildtype samples of the same age and the same run of the qPCR analysis [39]. Two additional housekeeping genes, *β-Actin* and *Rpl13a* were also tested. Calculation of *Rpl13a* expression using either *Ppia* or *β-Actin* as a housekeeping gene yielded marked differences between wildtype and *Npc1* ^-/-^ mice at 3 weeks of age (Table S2), making *Rpl13a* unsuitable as a housekeeping gene. *Ppia* and *β-Actin* were not differentially expressed between wildtype and *Npc1* ^-/-^ mice when calculated relative to each other (Table S2), and gave similar results when used as housekeeping genes for expression analyses of target genes. All qPCR data shown in the main body of the manuscript were standardized to *Ppia*. Data were derived from 8 to 15 mice of each genotype and each age.

### Immunoblotting

Snap-frozen tissue was homogenized in ice-cold HEPES buffer (10 mM HEPES, 1 mM EDTA, 1 mM EGTA, 1% Triton X-100, 0.5% Nonidet 40), with freshly added protease inhibitors (5 µg/ml leupeptin, 5 µg/ml aprotinin, 50 µM PMSF and 1 µM pepstatin) and phosphatase inhibitors (2 mM ortho-vanadate and 1 mM sodium fluoride) using a motor-driven pestle fitting into a microcentrifuge tube in a metal cooling block, followed by slow trituration through a syringe fitted with a 26 gauge needle. Protein content was determined by a bicinchoninic acid-based photometric assay (Thermo Fisher Scientific). Brain homogenates were separated by reducing SDS-PAGE and transferred to polyvinylidene fluoride membranes. The membranes were blocked in 5% skim milk powder in Tris-buffered saline with 5% Tween (TTBS, pH 7.4) and incubated with primary antibodies as indicated. When anti-phospho antibodies were used, membranes were blocked with TTBS containing 2% polyvinylpyrrolidone and the phosphatase inhibitors sodium orthovanadate and sodium fluoride. Secondary horseradish peroxidase-conjugated donkey anti-mouse, anti-rabbit, anti-chicken, or anti-goat antibodies (Jackson Immunoresearch, West Grove, PA) were diluted 1∶10,000 in TTBS, and detected by enhanced chemiluminescence. Actin was used as a loading control. Primary antibodies are listed in Table S3. Immunoblots were performed with samples from at least 6 mice of each genotype and age.

### Determination of mitochondrial DNA

DNA was isolated from 5-week old wildtype and *Npc1^-/-^* cerebellum by phenol-chloroform-isoamyl extraction as described [40]. The cytochrome *c* oxidase subunit I (*Mtco1*) gene of mitochondrial DNA and the *Ndufv1* gene of nuclear DNA were amplified by qPCR of 0.1 ng DNA using SYBR Green and primers as described [41] and listed in Table S1. Specificity of the amplification reaction was tested by melt curve analysis, agarose gel electrophoresis, and sequencing of the amplicon. Data were calculated by the Pfaffl method using *Ndufv1* as standard and *Mtco1* as target gene [39]. Data were derived from 10 mice of each genotype.

### ATP generation by isolated mitochondria

Cerebella, cerebral cortices and hippocampi were dissected from 3- or 7-week old wildtype and *Npc1* ^-/-^ mice and homogenized together in cold mitochondria isolation buffer (220 mM mannitol, 7 mM sucrose, 20 mM Hepes, pH 7.2, 1 mM EGTA, 0.1% bovine serum albumin (Fraction V, fatty acid free) and protease inhibitors. Mitochondria were isolated by differential centrifugation. ATP production was detected by the luciferase-luciferin method as described [42]. Briefly, mitochondria (100 or 50 µg) were incubated with energy substrates (1 mM pyruvate/1 mM malate or 5 mM succinate), ATP detection buffer (40 µM luciferin, 1/3000 luciferase (Biotium)) and ADP (300 µM) in Tris buffer (150 mM KCl, 25 mM Tris pH 7.4, 2 mM EDTA, 0.1% fatty acid free BSA, 10 mM KH_2_PO_4_, and 0.1 mM MgCl_2_) at 37°C. Luminescence was measured with a FLUOStar Optima plate reader (BMG Labtechnologies) for 20 readings at 1 s integration time. The rate of ATP generation was calculated as the luminescence increase during the measurement period of 20 s as luminescence (a.u.) per second per cell protein, and expressed as a percent of the average of wildtype samples of the same experiment. Addition of NaN_3_ (10 mM), rotenone (2 µg/ml), or antimycin A (50 µg/ml) reduced the luminescence increase during the measuring period to undetectable levels even in the presence of energy substrates (not shown). To verify mitochondrial polarization, an aliquot of each mitochondrial preparation was incubated with 2 µM nonyl acridine orange (Biotium, Hayward, CA) for 30 min at 37°C, washed, and analyzed for fluorescence at 488/520 nm excitation/emission.

### Neuronal glucose uptake

Primary cortical neurons were prepared from embryonic E17 wildtype and *Npc1* ^-/-^ mice essentially as described [43]. Neurons were grown in serum-free Neurobasal/MEM medium (1∶1 v/v), with 2% B27 supplement, 0.25 µM glutamate, and antibiotics for 10 days. Five days after plating, neurons were treated with 2.5 µM cytosine arabinofuranoside to prevent proliferation of glia cells. For measurement of glucose uptake, neurons were incubated with osmolarity-adjusted HEPES-buffered saline (10 mM HEPES, 124 mM NaCl, 3 mM KCl, 2 mM CaCl_2_, 1 mM MgCl_2_; osmolarity 270 to 290 mOsm), containing 2 mM glucose and 2 µCi/ml [^3^H]-deoxyglucose for 30 min. Cell-associated radioactivity was determined by scintillation counting and standardized to cell protein.

### Statistical Analysis

The analysis of the ^1^H-NMR spectra was performed using an in-house written code in MATLAB® 7.1 software (MathWorks, Natick, MA) [38], [44] and is described in detail in the Text S1. Briefly, spectra were segmented into bins and processed by principal component analysis (PCA) and by projection pursuit for exploratory data analysis (PPEDA) using Kurtosis as a projection index. Both types of analyses gave similar results regarding statistically significant differences between wildtype and *Npc1* ^-/-^ datasets. The results shown are from PPEDA. Following the initial exploratory data analysis, additional NMR spectra were acquired to achieve larger data sets for quantitative analysis of selected metabolites.

The Shapiro-Wilk test was used to verify the normality of the distribution of all other data sets [45]. All *Npc1* ^-/-^ and age-matched wildtype control data sets satisfied the conditions of a normal data distribution according to the Shapiro-Wilk Test for thresholds p = 0.01, p = 0.05 and p = 0.1, with the exception of the qPCR analysis of mRNA levels of *Gfap*. A student's two-tailed t-test was used for the comparison of normally distributed data from *Npc1* ^-/-^ and age-matched wildtype control, and the Mann-Whitney U test was used for the comparison of mRNA levels of *Gfap*. Statistical significance was assumed as p<0.05.

## Results

### Metabolomic differences between wildtype and *Npc1^-/-^* brain regions in early stages of NPC disease

**Figure 1:**
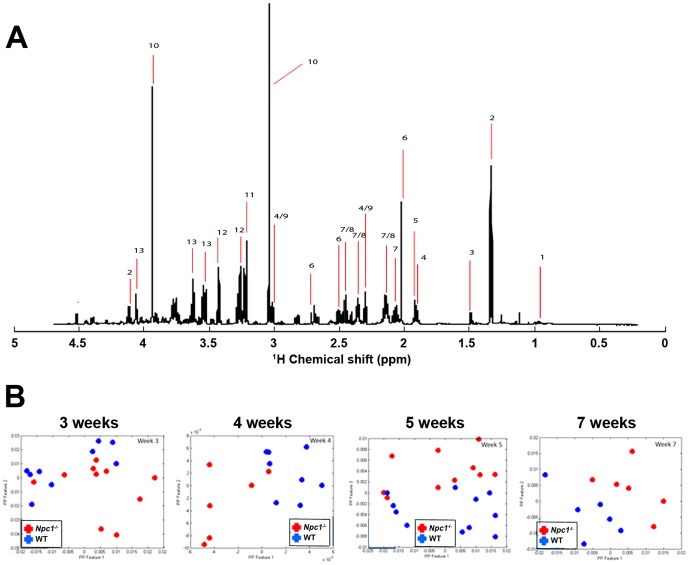
Metabolomics analysis of wildtype and *Npc1* ^-/-^ cerebellum. ^1^H-NMR spectroscopy of aqueous extracts from wildtype (WT) and *Npc1* ^-/-^ cerebellum. A) Sample spectrum. Peaks: 1) Branched chain amino and ketoacids, 2) Lactate, 3) Alanine, 4) GABA, 5) Acetate/acetyl-CoA, 6) NAA, 7) Glutamate, 8) Glutamine, 9) α-ketoglutarate, 10) Creatine, 11) Choline, 12) Taurine, and 13) myo-Inositol. B) PPEDA scores plots of sample sets of WT and *Npc1* ^-/-^ cerebellum at 3-, 4-, 5- and 7- weeks of age. The separation of blue (WT) and red (*Npc1* ^-/-^) data points indicates significant differences between WT and *Npc1* ^-/-^ sample sets at 4-, 5-, and 7- weeks of age.

To determine whether loss of NPC1 has consequences for energy metabolism in the brain, we first performed an unbiased metabolomics analysis of aqueous extracts of cerebellum, hippocampus and cerebral cortex tissue of wildtype and *Npc1* ^-/-^ mice by ^1^H-NMR spectroscopy. Brain tissue was analyzed at 3, 4, 5, and 7 weeks of age, representing pre-symptomatic (3 and 4 weeks), early symptomatic (5 weeks) and late stage (7 weeks) disease. Figure 1A shows a sample ^1^H-NMR spectrum of an aqueous extract of wildtype cerebellum. Spectra for all samples were normalized to unit sum and mean-centred prior to multivariate exploratory analysis via projection pursuit (PPEDA). PPEDA is similar to the more commonly used principal component analysis (PCA), but is less sensitive to outliers than PCA [46]–[48]. PCA employs linear combinations of the original variables of high-dimensional data to obtain new, fewer variables (termed principal components; PC) that account for the most variance, i.e., the most information, in the original data. Typically, only a few PCs are sufficient for a good representation of the data. The projections of the data onto the new coordinate system defined by these PCs are depicted in so-called scores plots as shown in Figure 1B. The scores plots allow an easy visual assessment of (dis)similarity among the data sets by the extent of clustering of data points derived from each data set. The scores plots resulting from our PCA analysis revealed significant differences between the metabolic profiles of wildtype and *Npc1* ^-/-^ cerebellum at 4, 5 and 7 weeks of age, but not at 3 weeks of age (Fig 1B). In the cerebral cortex, scores plots showed significant differences only for 7-week old mice (Figure S1A). No significant differences between genotypes were found in the metabolomic analysis of hippocampal samples (Figure S1B).

### Increased lactate and decreased acetate/acetyl-CoA levels in the cerebellum of 3-week old *Npc1^-/-^* mice

**Figure 2:**
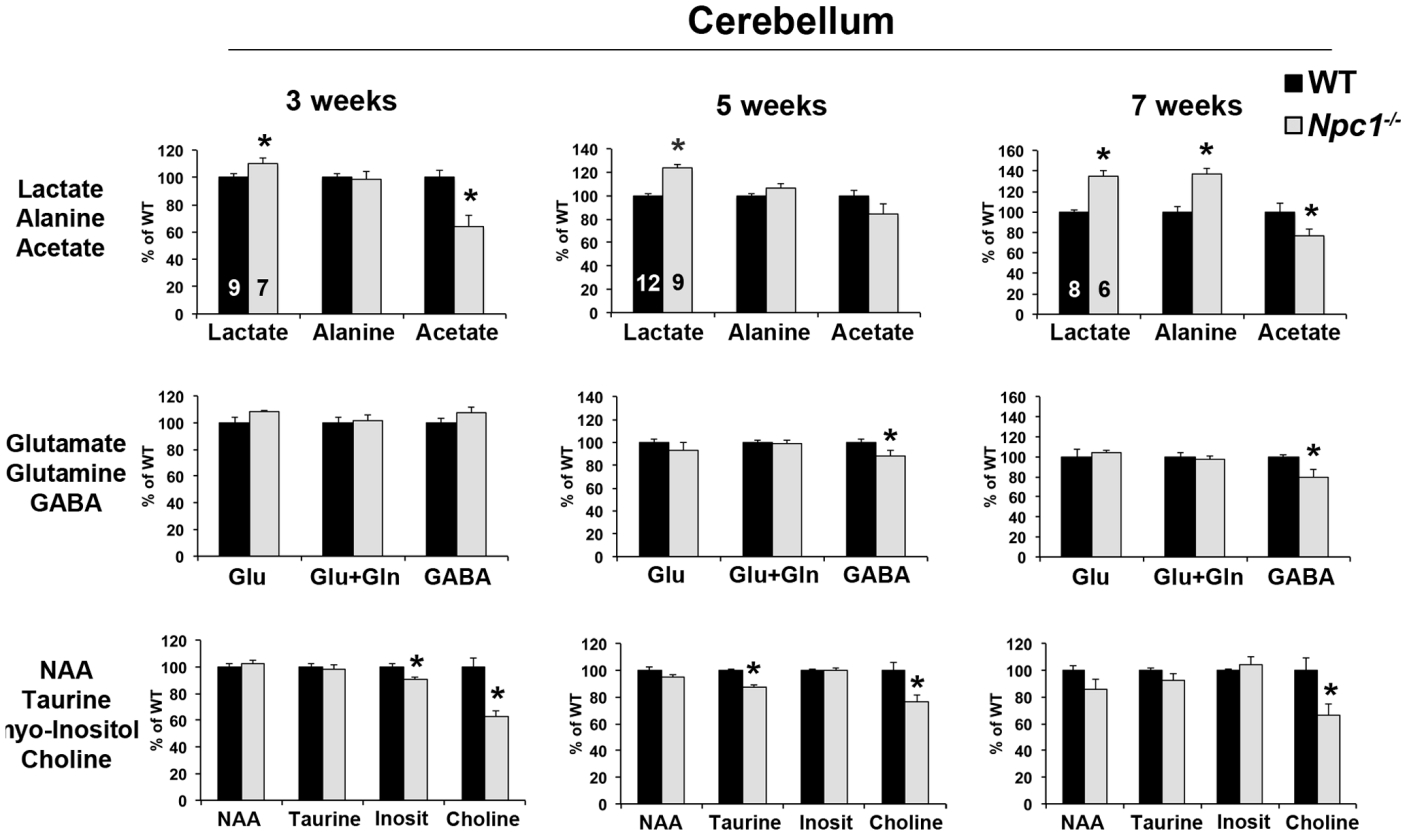
Altered energy metabolite levels in *Npc1* ^-/-^ cerebellum. Aqueous extracts of the cerebella from 3-, 4-, 5-, or 7-week old wildtype (WT) and *Npc1* ^-/-^ mice were analyzed by ^1^H-NMR spectroscopy. Spectra were deconvolved and integrated. Peak areas were standardized to total peak area. NAA: N-acetylaspartate. Inosit: myo-Inositol. Graphs in each column show data from the same set of WT and *Npc1* ^-/-^ mice of one age. The small numbers inside the bars for lactate indicate the number of mice in each group. Data are expressed as percent of the average of WT samples of the same age. Shown are the mean ± SEM. * p<0.05, *Npc1* ^-/-^ vs. WT.

The scores plots alone can only show whether wildtype and *Npc1* ^-/-^ brain regions differed in their overall metabolic profile, but they do not yield information about particular metabolites. On the other hand, the levels of specific metabolites could be different between the genotypes without causing a significant difference in the overall metabolic profile analyzed by PCA. We therefore deconvolved and integrated all spectra and determined the peak areas for all metabolites that were clearly distinguishable in the spectra. The peak areas were standardized to the total peak area to reflect the relative levels of energy metabolites in the aqueous extract. The most striking differences between wildtype and *Npc1* ^-/-^ brain extracts were found in lactate and acetate/acetyl-CoA, which are the products of pyruvate reduction in anaerobic glycolysis and of oxidative decarboxylation of pyruvate during aerobic glycolysis, respectively. The resonance frequencies of the CH_3_-group of acetate and acetyl-CoA overlap near 1.92 ppm, and can therefore not be distinguished in the ^1^H-NMR spectra (Fig 1). Lactate levels were significantly higher in *Npc1* ^-/-^ cerebellum and cerebral cortex compared to wildtype, and increased with disease progression in *Npc1* ^-/-^ cerebellum (Fig 2 and Figure S2). In contrast, acetate/acetyl-CoA levels were markedly decreased in the cerebellum and cerebral cortex of *Npc1* ^-/-^ mice aged 3 or 7 weeks. In *Npc1* ^-/-^ hippocampus, acetate/acetyl-CoA levels were significantly decreased at both 3 and 5 weeks of age; however, lactate levels were unaltered (Figure S3). Alanine, which is catabolized to pyruvate by transamination, was increased in the cerebellum of 7-week old *Npc1* ^-/-^ mice (Fig 2).

Our metabolomics analysis also revealed a decrease in the levels of the inhibitory neurotransmitter gamma-aminobutyric acid (GABA) in *Npc1* ^-/-^ cerebellum at 5 and 7 weeks of age and in *Npc1* ^-/-^ cerebral cortex at 7 weeks of age, while glutamate levels were not altered (Fig 2 and Figure S2). Hippocampal *Npc1* ^-/-^ samples showed a slight decrease in GABA levels at 3 weeks and an increase in glutamate levels at 5 weeks of age (Figure S3). Thus, the neurotransmitter balance appeared to be shifted towards increased excitatory or decreased inhibitory neurotransmission in all brain regions analyzed. The resonance frequencies of glutamine and glutamate largely overlap, except for a region between 2.04 to 2.07 ppm that corresponds mostly to glutamate. Peaks in this region were integrated for quantification of glutamate, and regions with overlapping resonance frequencies were quantified as the sum of glutamate and glutamine (Glu + Gln). There were no differences between genotypes in these peak ranges, suggesting that glutamine levels were not altered in *Npc1* ^-/-^ cerebellum (Fig 2).

In spite of the known Purkinje cell loss in the cerebellum of older *Npc1* ^-/-^ mice, the levels of the neuron-derived metabolite N-acetylaspartate (NAA) were not different between genotypes, with only a non-significant (p = 0.08) decrease in *Npc1* ^-/-^ cerebellum at 7 weeks of age (Fig 2). The levels of the osmolytes taurine and myo-inositol were slightly decreased in *Npc1* ^-/-^ cerebellum at 5 and 3 weeks of age, respectively (Fig 2). The lack of alterations in myo-inositol content at more advanced stages of the disease was somewhat surprising, since the levels of the glia-derived myo-inositol commonly increase during glial activation and/or proliferation. Creatine levels remained unaltered in all samples (not shown). All brain areas of *Npc1* ^-/-^ mice 3 weeks of age and older showed a striking decrease in their levels of choline (Fig 2), which plays a key role in brain development as an important precursor of one-carbon metabolism and phospholipid synthesis [49].

### Alterations in glucose and pyruvate metabolism in *Npc1^-/-^* cerebellum

**Figure 3:**
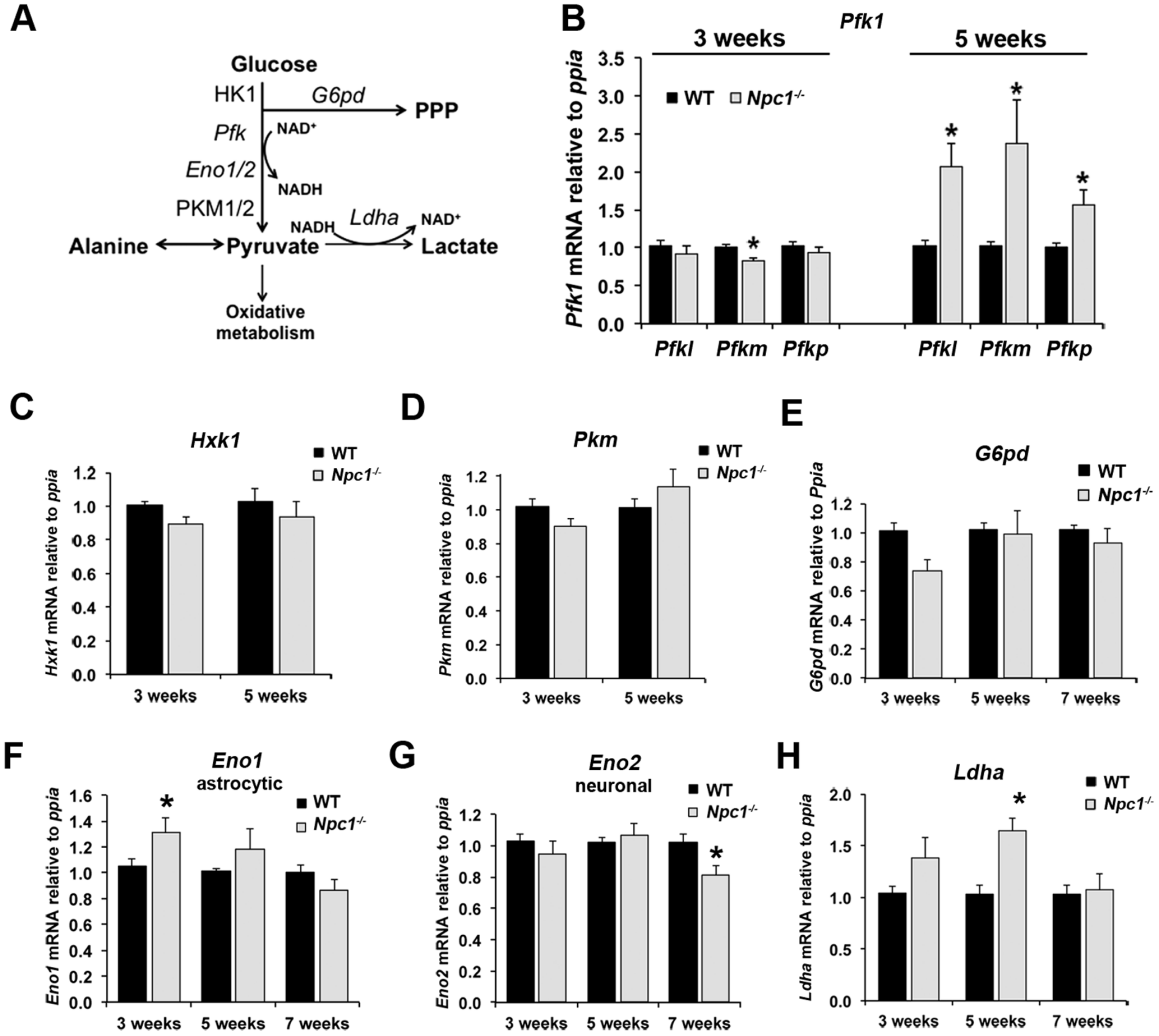
Increased expression of glycolytic enzymes in *Npc1* ^-/-^ cerebellum at 5 weeks of age. A) Schematic representation of glycolysis and connected pathways showing mRNA (italics) and proteins analyzed in this study. PPP: pentose phosphate pathway. B – H) Cerebellar RNA extracts were prepared from 3-, 5- or 7-week old wildtype (WT) and *Npc1* ^-/-^ mice, and mRNA levels of the target genes were analyzed by qPCR using cyclophilin (*Ppia*) as housekeeping gene. B) Phosphofructokinase isoforms (*Pfkl*, *Pfkm*, *Pfkp*), *C*) Hexokinase 1 (*Hxk1*); D) Pyruvate Kinase Type M *(Pkm)*, E) astrocytic alpha-Enolase (*Eno1*), F) neuronal Enolase (*Eno2*), G) Lactate Dehydrogenase (*Ldha*), H) Glucose-6-phosphate dehydrogenase (*G6pd*). Data are shown as mean ± SEM. * p<0.05, *Npc1* ^-/-^ vs. WT.

The increased lactate levels suggested that anaerobic glycolysis was increased in the *Npc1* ^-/-^ brains, while the decrease in acetate/acetyl-CoA levels pointed to a decrease in the oxidative decarboxylation of pyruvate to acetyl-CoA for oxidative metabolism of glucose. Given that there is limited fatty acid oxidation in the brain [50], the majority of acetyl-CoA found in the brain is derived from pyruvate. To investigate potential alterations in glucose metabolism in NPC1-deficient brain tissue, we first measured mRNA expression levels of several key enzymes of glycolysis in wildtype and *Npc1* ^-/-^ cerebellum by qPCR. A schematic overview of glycolysis and its enzymes is shown in Fig 3A. The main rate-determining step of glycolysis is catalyzed by phosphofructokinase-1 (*Pfk*). At 3 weeks of age, we found no differences in the mRNA levels of two of the three PFK1 isoforms (*Pfkl* and *Pfkp*), and decreased mRNA levels of the *Pfkm* isoform in *Npc1* ^-/-^ cerebellum compared to wildtype (Fig 3B). However, at 5 weeks of age, mRNA levels of all three isoforms of PFK1 were markedly increased in *Npc1* ^-/-^ cerebellum (Fig 3B), suggesting that at this stage of the disease, glycolysis is upregulated on the gene expression level. In contrast, the mRNA levels of hexokinase 1 (*Hxk1*) and pyruvate kinase type M (*Pkm*), which catalyze the two additional exergonic, highly regulated steps of glycolysis, were unchanged in *Npc1* ^-/-^ cerebellum at either 3 or 5 weeks of age (Fig 3C and D). Glucose-6-phosphate dehydrogenase (*G6pd*), which catalyzes the rate-limiting entrance of glucose-6-phosphate into the pentose-phosphate pathway, appeared decreased in *Npc1* ^-/-^ cerebellum at 3 weeks of age, but this decrease was not significant (p = 0.07; Fig 3E). To gain insight into the relative contributions of neurons and astrocytes, we measured mRNA levels of enolase, which is expressed in different isoforms in the two cell types [51]. While mRNA levels of astrocytic alpha-enolase (*Eno1*) were increased in *Npc1* ^-/-^ cerebellum as early as 3 weeks of age, mRNA levels of neuron-specific enolase (*Eno2*) were unaltered at early stages of the disease and decreased in *Npc1* ^-/-^ cerebellum at 7 weeks of age (Fig 3F and G). In addition, mRNA levels of the mostly astrocytic isoform of lactate dehydrogenase (*Ldha*), which catalyzes the production of lactate in the last step of anaerobic glycolysis, were significantly increased in *Npc1* ^-/-^ cerebellum at 5 weeks of age (Fig 3H), in line with the increased lactate levels observed by NMR-spectroscopy (Fig 2). Together, these findings indicated differences in the transcriptional regulation of glucose metabolism, and a possible upregulation of glycolysis at the early symptomatic stage of the disease, in particular in astrocytes.

**Figure 4:**
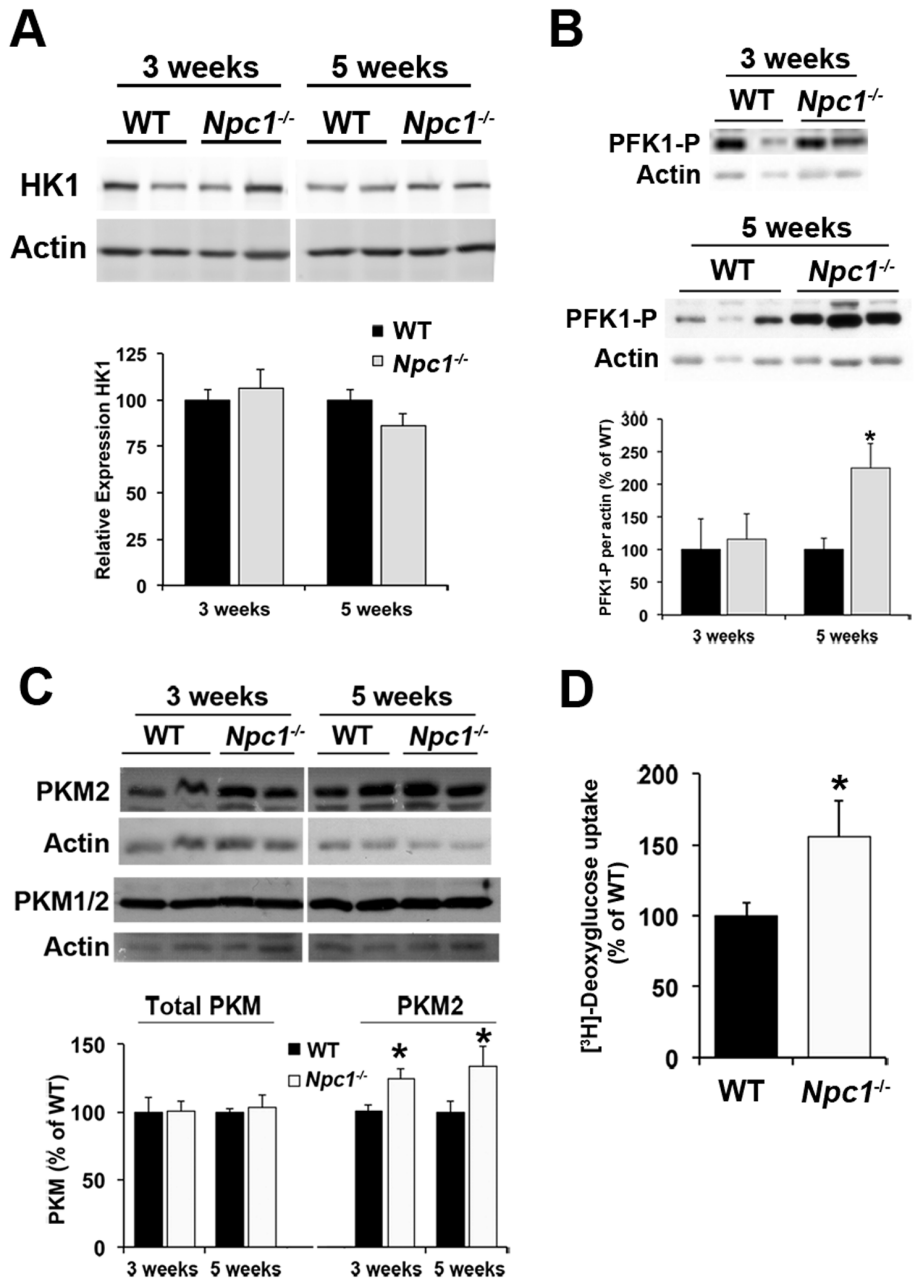
Increased phosphofructokinase levels in *Npc1* ^-/-^ cerebellum at the early symptomatic stage of the disease. A to C) Cerebellar tissue homogenates were prepared from 3- or 5-week old wildtype (WT) and *Npc1* ^-/-^ mice and analyzed by immunoblotting using antibodies against hexokinase 1 (HK1; panel A), phosphofructokinase 1 (PFK1P; panel B), both isoforms 1 and 2 of pyruvate kinase type M (PKM1/2; panel C), and isoform 2 of PKM alone (PKM2, panel C) and actin as a loading control (all panels A to C). Bar graphs show the ratio of the band intensities of the proteins of interest to band intensity of actin immunoreactivity on the same membrane and are expressed as percent of the average of WT samples on the same membrane. D) Primary cortical neurons from embryonic WT and *Npc1* ^-/-^ mice were incubated for 30 min in HEPES-buffered saline with [^3^H]-2-deoxyglucose and 2 mM glucose. Shown is the cell-associated radioactivity standardized to cell protein and expressed as percent of WT neurons of the same experiment (3 independent experiments in duplicate). All bar graphs show the mean ± SEM. * p<0.05, *Npc1* ^-/-^ vs. WT.

Immunoblot analyses of HK1, PFK1 and PKM protein levels in *Npc1* ^-/-^ cerebellum showed a pattern similar to the mRNA analysis. While HK1 levels were not altered (Fig 4A), protein levels of PFK1-P, which is highly expressed in cerebellum [52], were increased in *Npc1* ^-/-^ cerebellum at 5 weeks but not at 3 weeks of age (Fig 4B), further pointing to an increase in glycolysis in cerebellum at the early symptomatic stage of NPC disease. Total PKM protein levels were unaltered, however, the protein levels of the PKM2 isoform were increased in *Npc1* ^-/-^ cerebellum at 3 and 5 weeks of age (Fig 4C).

The observed increase in lactate levels, and increased levels of *Eno1* and *Ldha* mRNA suggested increases in astrocytic glycolysis in *Npc1* ^-/-^ cerebellum. To investigate whether glycolysis may also be altered in *Npc1* ^-/-^ neurons, we measured the uptake of radiolabeled 2-deoxyglucose into primary wildtype and *Npc1* ^-/-^ cortical neurons after 9 days of culture. 2-Deoxyglucose is phosphorylated by hexokinase but not metabolized further, therefore its accumulation in the cells reflects glucose uptake and phosphorylation for glycolysis and pentose phosphate pathway [53]. *Npc1* ^-/-^ neurons had significantly higher 2-deoxyglucose uptake than wildtype primary neurons (Fig 4D), suggesting that glucose uptake and glycolysis can increase in *Npc1* ^-/-^ neurons.

### Decreased oxidative metabolism of pyruvate in *Npc1^-/-^* cerebellum

**Figure 5:**
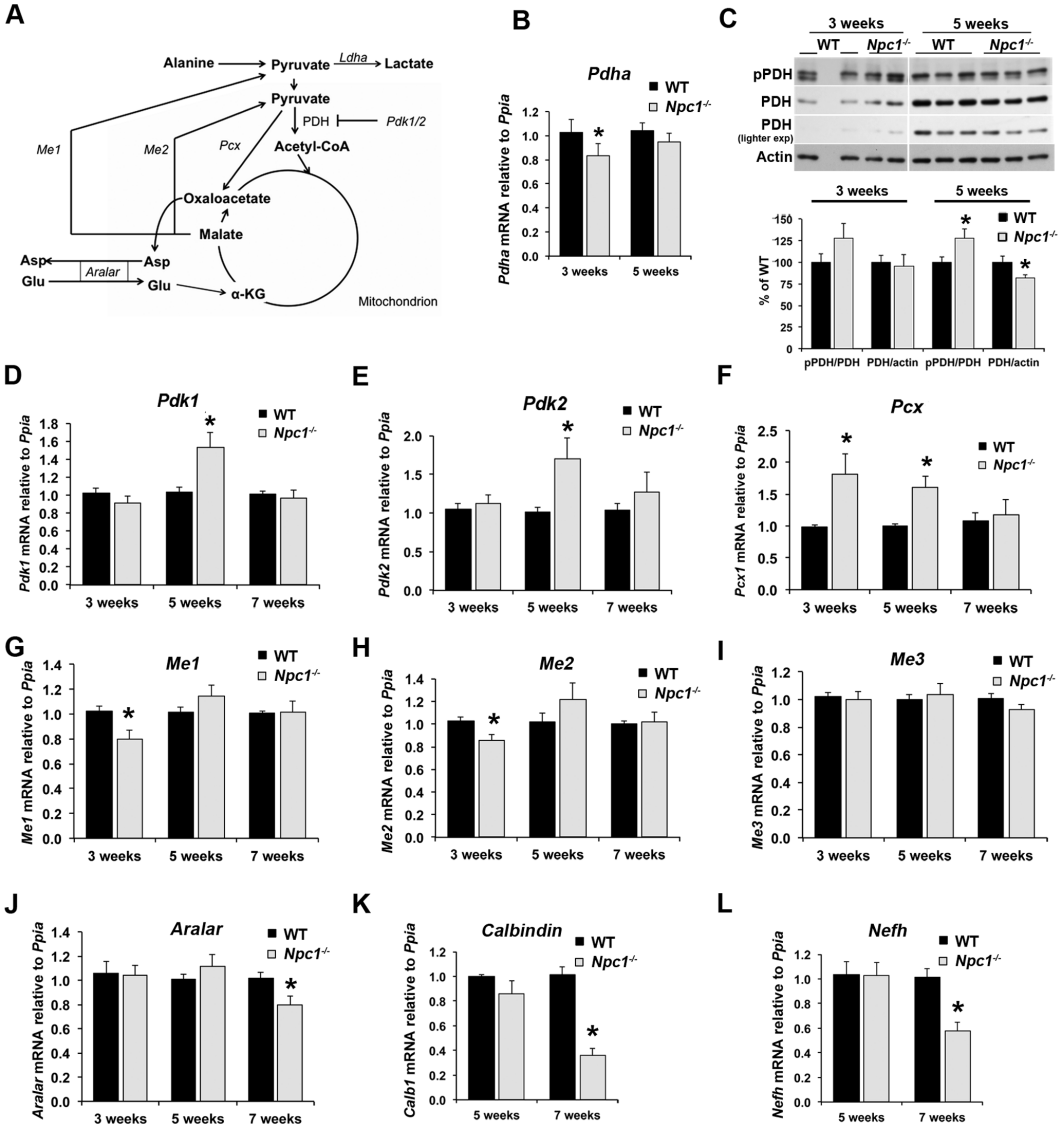
Altered expression of pyruvate metabolism-related genes in *Npc1* ^-/-^ cerebellum. Cerebellar tissue homogenates and RNA were prepared from 3-, 5-, or 7-week old wildtype (WT) and *Npc1* ^-/-^ mice. A) Schematic representation of pathways of pyruvate metabolism showing mRNA (italics) and proteins analyzed in this study. The grey shading represents the mitochondrial compartment. B) mRNA levels of pyruvate dehydrogenase subunit A (*Pdha*) analyzed by qPCR standardized to *Ppia*. C) Immunoblots of tissue homogenates using antibodies against phospho-PDH and PDH, and actin as loading control. The bar graph shows the ratio of phospho-PDH per PDH, and PDH per actin as percent of the average of WT samples on the same membrane. D – L) mRNA levels of pyruvate dehydrogenase kinase 1 and 2 (*Pdk1* and *Pdk2*), pyruvate carboxylase (*Pcx*), cytosolic, mitochondrial, and neuronal mitochondrial malic enzymes (*Me1, Me2, Me3*), Aralar, Calbindin (*Calb1*), and neurofilament heavy polypeptide (*Nefh*) analyzed by qPCR standardized to *Ppia*. All bar graphs show the mean ± SEM. * p<0.05, *Npc1* ^-/-^ vs. WT.

Pyruvate derived from glycolysis can enter several different pathways, including the reversible transamination with alanine or the reduction to lactate. Pyruvate is also transported into mitochondria, where it can undergo carboxylation to oxaloacetate or oxidative decarboxylation to acetyl-CoA, which can itself be further oxidized in the citric acid cycle (schematic overview shown in Fig 5A). The increased lactate to acetate/acetyl-CoA ratio observed by NMR spectroscopy in *Npc1* ^-/-^ brain at all ages suggested that mitochondrial oxidative metabolism of pyruvate was decreased, and that the cytosolic reduction of pyruvate to lactate was increased to supply NAD^+^ for glycolysis.

To investigate potential alterations in the mitochondrial metabolism of pyruvate in *Npc1* ^-/-^ cerebellum, we measured the mRNA levels of several key enzymes of pyruvate metabolism and of the malate aspartate transporter Aralar 1, which is part of a mitochondrial shuttle for the indirect transfer of NAD^+^ into the cytosol. The oxidative decarboxylation of pyruvate to acetyl-CoA is catalyzed by the pyruvate dehydrogenase complex PDH in the mitochondrial matrix. Expression analysis of PDH showed lower mRNA levels of the *Pdha* subunit in *Npc1* ^-/-^ cerebellum at 3 weeks of age (Fig 5B). Moreover, immunoblot analysis revealed a decrease in the total levels of PDH in *Npc1* ^-/-^ cerebellum at 5 weeks of age, and an increase in the levels of phosphorylated PDH at 3 and 5 weeks of age (Fig 5C). PDH is inhibited by phosphorylation; therefore, these findings strongly suggested a decrease in the oxidation of pyruvate to acetyl-CoA, in line with the decreased acetate/acetyl-CoA levels observed by NMR spectroscopy (Fig 2). The increased mRNA levels of PDH kinases 1 and 2 in *Npc1* ^-/-^ cerebellum at 5 weeks of age (Fig 5D and E; *Pdk1* and *Pdk2*) suggested a longer-term increase in PDH phosphorylation on the gene expression level in *Npc1* ^-/-^ cerebellum at this age. Given that PDK1 and PDK2 are more prevalent in neurons and astrocytes, respectively [54], these alterations in PDH likely occurred in both cell types. Cerebellar mRNA levels of pyruvate carboxylase (*Pcx*), which converts pyruvate to oxaloacetate only in astrocytes, were increased in 3- and 5-week old *Npc1* ^-/-^ mice (Fig 5F), while mRNA levels of cytosolic malic enzyme (*Me1*), which catalyzes the interconversion of pyruvate and malate mainly in the direction of pyruvate synthesis, were decreased in *Npc1* ^-/-^ cerebellum at 3 weeks of age (Fig 5G). Mitochondrial malic enzyme 2 (*Me2*) mRNA levels were also decreased in *Npc1* ^-/-^ cerebellum at 3 weeks of age (Fig 5H), while mRNA levels of the mainly neuronal, mitochondrial malic enzyme 3 (*Me3*) and the malate aspartate shuttle aralar (*Aralar1*) were unchanged, except at 7 weeks of age (Fig 5I and J). The decreased mRNA levels of neuronal *Eno2* and *Aralar1* in *Npc1* ^-/-^ cerebellum at 7 weeks of age (Fig 3G and 5J) likely reflected Purkinje neuron death, as mRNA levels of the Purkinje cell marker calbindin (*Calb1*) and of the neuron-specific neurofilament heavy peptide (*Nefh*) were decreased by 60% and 25% respectively at this age (Fig 5K and L).

Together, these findings demonstrated that alterations in cerebellar glucose metabolism (upregulation of *Eno1* and *Pcx* mRNA, higher PKM2 protein, decreased *Pdha*, *Me1* and *Me2* mRNA levels, increased lactate and decreased acetate/acetyl-CoA levels) developed early in NPC disease, and could already be detected in 3-week old, pre-symptomatic *Npc1* ^-/-^ mice. Dysregulation of gene expression and of the metabolic profile of *Npc1* ^-/-^ cerebellum was even more pronounced at 5 weeks, when symptoms become apparent in *Npc1* ^-/-^ mice.

### Mitochondrial alterations in *Npc1^-/-^* brain

**Figure 6:**
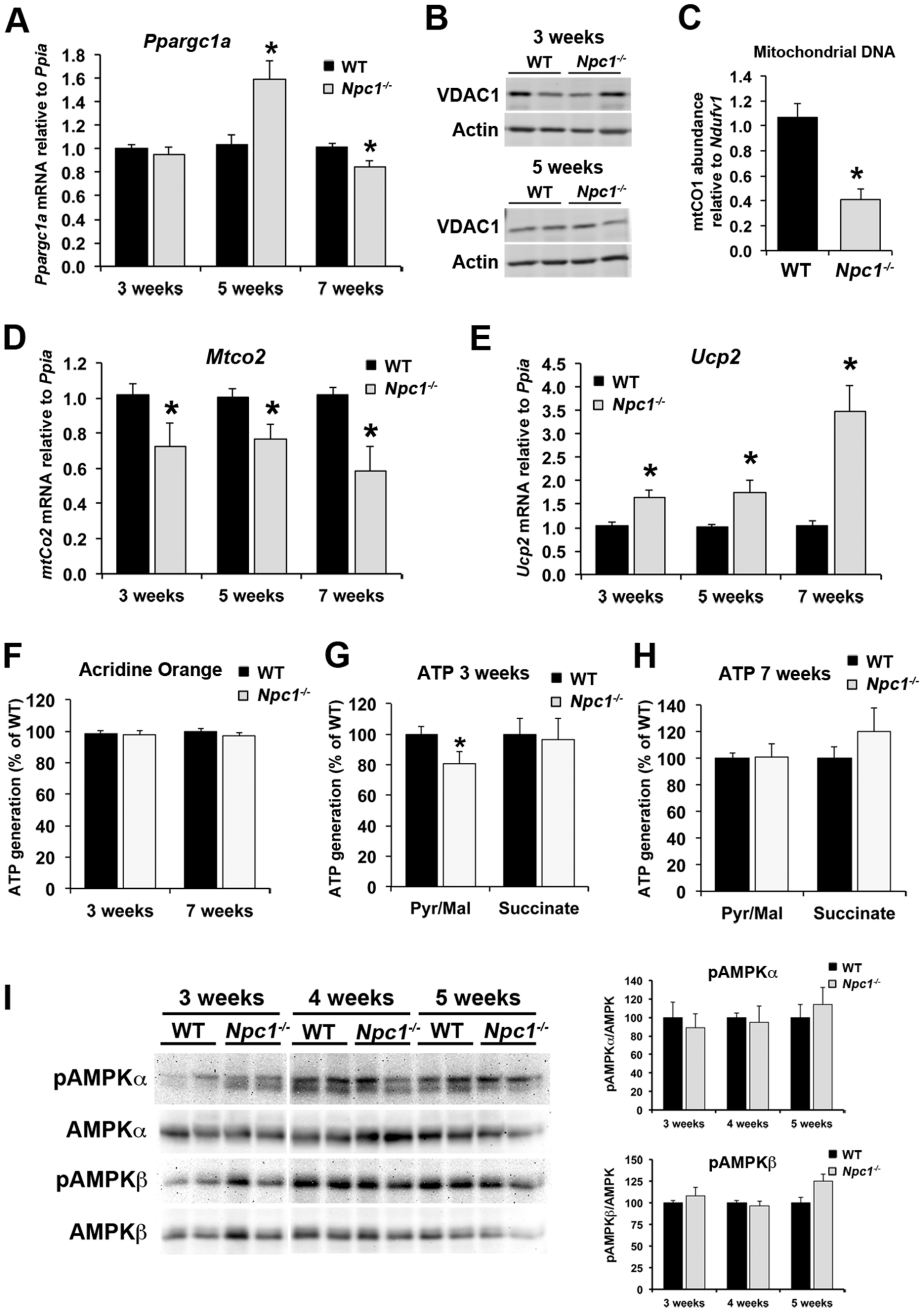
Mitochondrial alterations in *Npc1* ^-/-^ cerebellum. A) Cerebellar RNA was prepared from 3-, 5- or 7-week old WT and *Npc1* ^-/-^ mice. Target gene mRNA levels were measured by qPCR using primers against PGC1α (*Ppargc1a*) standardized to *Ppia*. B) Immunoblots of cerebellar tissue homogenates from 3- or 5-week old wildtype (WT) and *Npc1* ^-/-^ mice probed with antibodies against VDAC1 and actin as loading control. C) Genomic DNA was prepared from cerebella of 5-week old WT and *Npc1* ^-/-^ mice. Mitochondrial DNA was determined by qPCR using primers against mitochondrial cytochrome c oxidase subunit 1 (*Mtco1*) DNA per nuclear *Ndufv1* DNA, and standardized to WT. D and E) Cerebellar RNA was prepared as in panel A. Target gene mRNA levels were measured by qPCR using primers against mitochondrially encoded subunit 2 of cytochrome c oxidase (*Mtco2*) or uncoupling protein 2 (*Ucp2*) standardized to *Ppia*. F to H) Mitochondria were isolated by differential centrifugation from combined homogenates of the cerebella, hippocampi and cerebral cortices of wildtype (WT) and *Npc1* ^-/-^ mice at 3 and 7 weeks of age. F) Mitochondria were stained with nonyl acridine orange to verify the maintenance of their membrane potential. G and H) The rate of ATP generation was measured in the presence of 1 mM pyruvate, 1 mM malate and 300 µM ADP (Pyr/Mal) or in the presence of 5 mM succinate and 300 µM ADP as luminescence generated by the ATP-dependent D-luciferin/luciferase reaction as luminescence per second per protein. F) Mitochondria isolated from 3-week old mice. G) Mitochondria isolated from 7-week old mice. Data shown in panels F – H) are expressed as percent of WT of the same experiment, and are derived from three independent experiments each with two independent mitochondria preparations of each genotype and each age. I) Cerebellar tissue homogenates were prepared from 3-, 4- or 5-week old WT and *Npc1* ^-/-^ mice and analyzed by immunoblotting using antibodies against phospho-AMPKα (Thr172), AMPKα, phospho-AMPKβ (Ser108), AMPKβ, and actin as loading control. Bar graphs show the ratio of band intensities of phosphorylated to total AMPK expressed as percent of the average of WT on the same membrane. All bar graphs show the mean ± SEM. * p<0.05, *Npc1* ^-/-^ vs. WT.

Next, we asked whether the upregulation of glycolytic enzymes and the increased lactate production in *Npc1* ^-/-^ cerebellum were a response to decreased mitochondrial abundance and/or mitochondrial defects, either of which would lower glucose oxidation and decrease mitochondrial ATP production. A key regulator of mitochondrial biogenesis and function is the peroxisome proliferator-activated receptor gamma coactivator 1 alpha (PGC-1α), which interacts with a variety of transcription factors involved in energy metabolism and mitochondrial function to increase mitochondrial biogenesis. PGC-1α mRNA (*Ppargc1a*) levels were not altered in *Npc1* ^-/-^ cerebellum at 3 weeks, but were markedly increased compared to wildtype at 5 weeks of age, and decreased at 7 weeks of age (Fig 6A), suggesting that at 5 weeks of age, the cellular response to increase mitochondrial biogenesis was activated in *Npc1* ^-/-^ cerebellum. We therefore determined the levels of a mitochondrial marker protein, the voltage dependent anion channel (VDAC1), and levels of mitochondrial DNA as measures for mitochondrial abundance. While VDAC1 protein levels remained unaltered throughout disease progression, mitochondrial DNA levels were substantially decreased in the cerebella of 5-week old *Npc1* ^-/-^ mice (Fig 6B and C), suggesting a loss of normal mitochondria even though *Ppargc1a* mRNA levels were increased. Additional alterations in mitochondria were seen in a decrease in mRNA levels of the mitochondria-encoded subunit 2 of cytochrome c oxidase (*Mtco2*) and a marked increase of mRNA levels of uncoupling protein 2 (*Ucp2*) in *Npc1* ^-/-^ cerebellum at all ages (Fig 6D and E). These observations suggested alterations in mitochondrial abundance, quality and electron transport chain components that could affect energy homeostasis in *Npc1* ^-/-^ cerebellum.

To investigate whether oxidative phosphorylation may be impaired in *Npc1* ^-/-^ brain, we measured ATP production by mitochondria isolated from wildtype and *Npc1* ^-/-^ brain at 3 and 7 weeks of age. Mitochondrial polarization was verified with acridine orange staining (Fig 6F). In the presence of pyruvate and malate as energy substrates, mitochondria isolated from *Npc1* ^-/-^ brain at 3 weeks produced less ATP than mitochondria from wildtype brain, in line with a decrease in PDH activity and impaired oxidative phosphorylation in *Npc1* ^-/-^ brain. On the other hand, in the presence of succinate, ATP generation was unchanged (Fig 6G), which may suggest that most components of the electron transport chain remained relatively unaffected in mitochondria from *Npc1* ^-/-^ brain. Interestingly, mitochondria isolated from wildtype and *Npc1* ^-/-^ brain at 7 weeks of age showed no differences in ATP generation (Fig 6H). While we cannot explain why isolated mitochondria from *Npc1* ^-/-^ brain at later stages of the disease appear to be normal in this assay, the findings reflect the lack of differences between genotypes at 7 weeks of age in most of our metabolic gene expression analyses (Fig 3 and 5). It is important to note, however, that assays using isolated mitochondria cannot fully reflect mitochondrial function in the brain. Isolation procedures and the assay environment strongly influence the measurements, and alterations in mitochondrial function in the brain are not necessarily recapitulated by isolated mitochondria.

To further investigate energy homeostasis in *Npc1* ^-/-^ cerebellum, we determined the levels of phosphorylated AMP-activated kinase (AMPK) by immunoblotting. A drastic loss of oxidative phosphorylation capacity would be expected to have a significant impact on energy homeostasis and likely lead to phosphorylation and activation of AMPK. However, levels of phosphorylated AMPK were unchanged in *Npc1* ^-/-^ cerebellum at 3, 4, and 5 weeks of age (Fig 6I), suggesting that there was no significant overall energy deficiency, possibly due to increased glycolytic ATP production.

### Activation of antioxidant defense systems in *Npc1^-/-^* cerebellum

**Figure 7:**
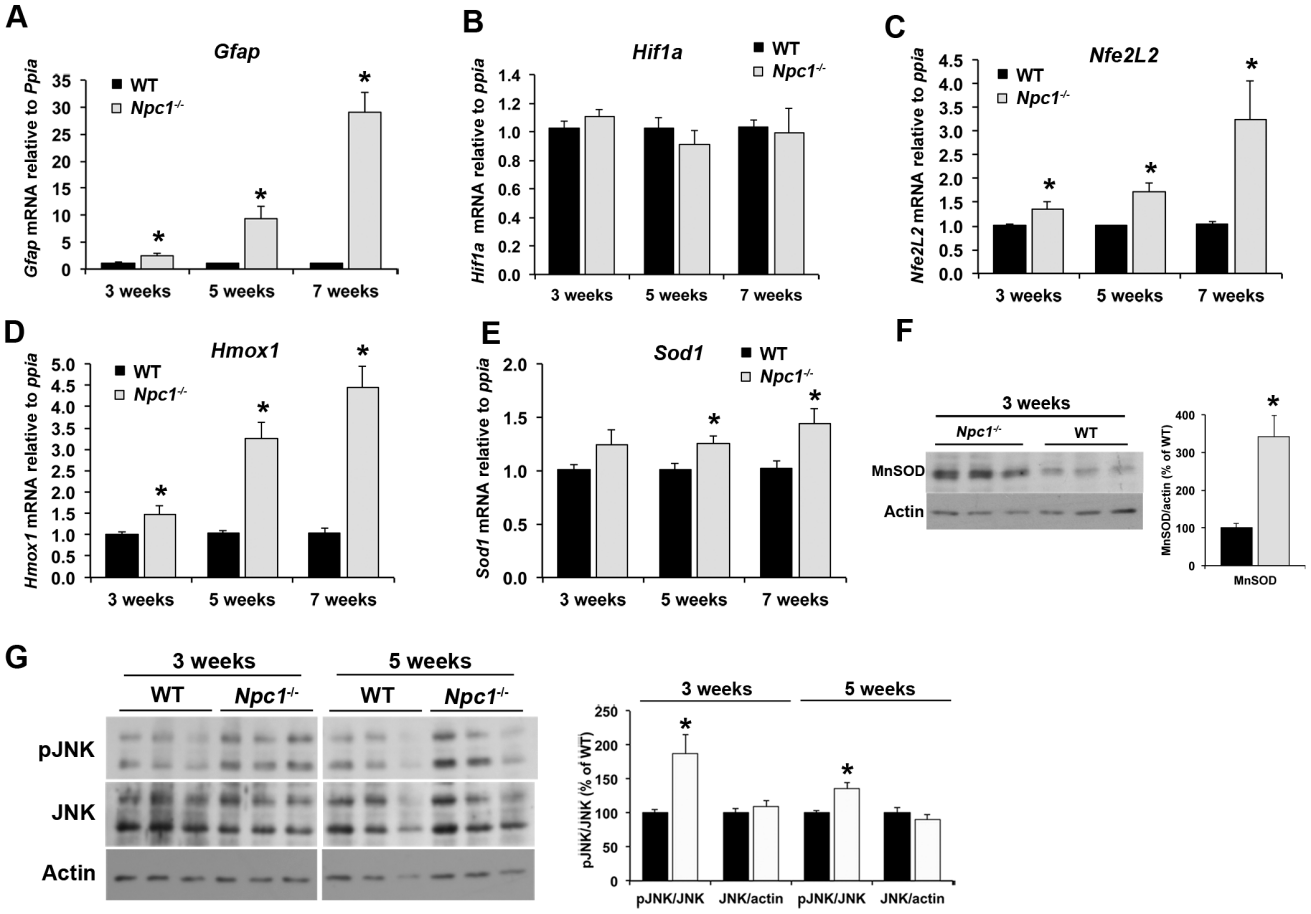
Early activation of antioxidant response genes in *Npc1* ^-/-^ cerebellum. A – E) Cerebellar RNA was prepared from 3-, 5-, and 7-week old wildtype (WT) and *Npc1* ^-/-^ mice. Target gene mRNA levels were analyzed by qPCR using primers against glial fibrillary acidic protein (*Gfap*), hypoxia-inducible factor 1alpha (*Hif1a*), nuclear factor erythroid 2-related factor 2 (*Nfe2l2*), heme oxygenase 1 (*Hmox1*), and superoxide dismutase 1 (*Sod1*), standardized to *Ppia*. F) Immunoblot analysis of tissue homogenates of cerebellum from 3-week old WT and *Npc1* ^-/-^ mice using antibodies against superoxide dismutase (MnSOD) and actin as a loading control. Bar graphs show the ratio of band intensities of MnSOD to actin expressed as percent of the average of WT on the same membrane. G) Immunoblot analysis of tissue homogenates of cerebellum from 3- or 5-week old WT and *Npc1* ^-/-^ mice using antibodies against phospho-JNK and JNK and actin as a loading control. Bar graphs show the ratio of band intensities of phosphorylated to total JNK, and the ratio of JNK to actin expressed as percent of the average of WT on the same membrane. All bar graphs show the mean ± SEM. * p<0.05, *Npc1* ^-/-^ vs. WT.

Next, we measured mRNA levels of several transcription factors or known target genes associated with stress responses. Glial fibrillary acidic protein (*Gfap*), a marker for astrocyte activation, was significantly increased in *Npc1* ^-/-^ brain as early as 3 weeks of age (Fig 7A), in accordance with previous reports [55]–[57]. Glycolytic gene expression and anaerobic glycolysis are also induced by activation of hypoxia-induced factor 1α (HIF-1α, *Hif1a*) [58]. However, *Hif1α* mRNA levels were not different between genotypes (Fig 7B), and we were unable to reliably detect HIF-1α by immunoblotting in either wildtype or *Npc1* ^-/-^ brain homogenates. In contrast, mRNA levels of the nuclear factor E2 related factor 2 (Nrf2, *Nfe2l2*), which is a key element of a protective response to oxidative stress, and of one of its target genes, heme oxygenase 1 (HO1, *Hmox1*), were markedly higher in *Npc1* ^-/-^ cerebellum beginning at 3 weeks of age, and increased with disease progression (Fig 7C and D). The early activation of antioxidant responses was also apparent in the increased mRNA levels of superoxide dismutase 1 (*Sod1*) and the increased protein levels of superoxide dismutase 2 (MnSOD) in *Npc1* ^-/-^ compared to wildtype cerebellum (Fig 7E and F). Moreover, we observed higher levels of phosphorylation of both isoforms of the c-jun N-terminal kinase (JNK) in *Npc1* ^-/-^ cerebellum at 3 and 5 weeks of age (Fig 7G). Phosphorylation and activation of JNK is a common response to oxidative stress and a wide range of other stress signals, suggesting that oxidative stress increases very early during NPC disease pathogenesis.

## Discussion

**Figure 8:**
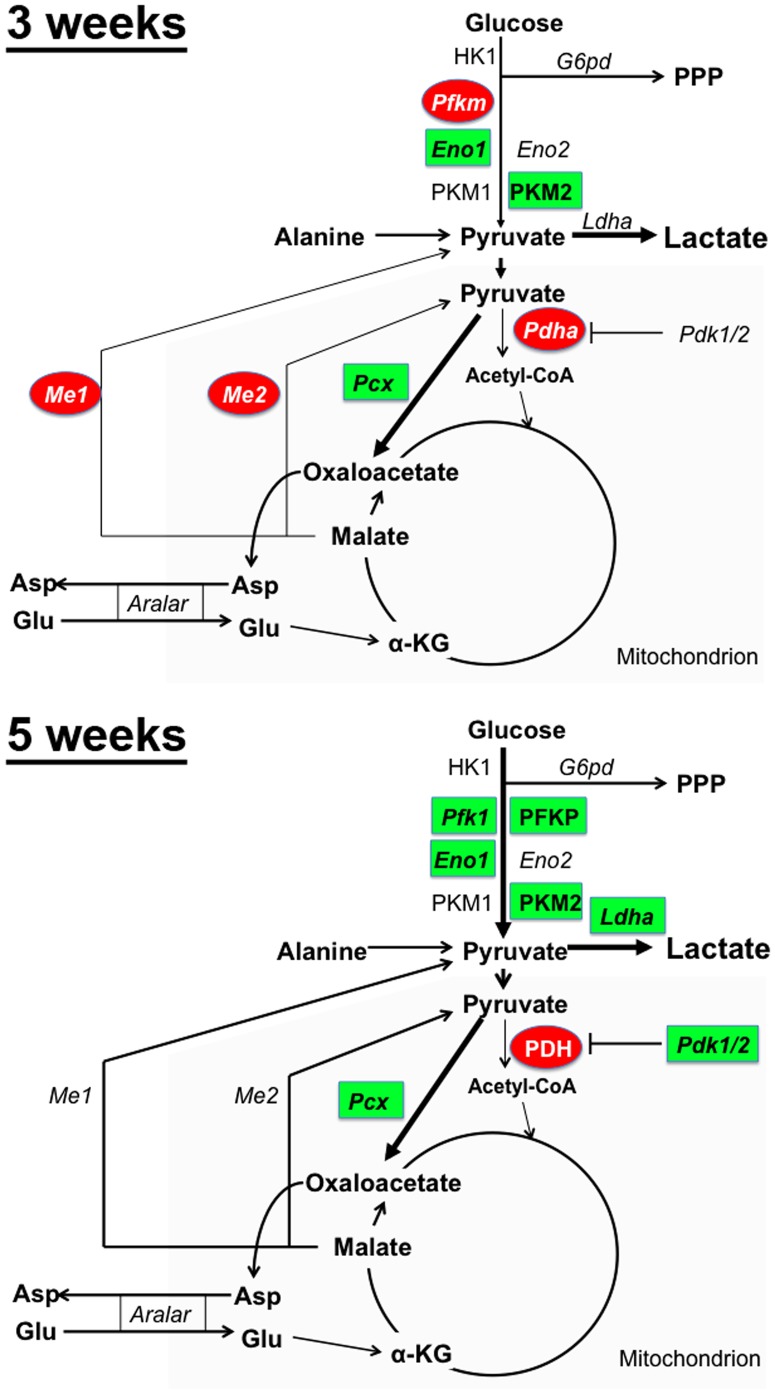
Overview of the alterations in gene and protein expression in *Npc1* ^-/-^ cerebellum and the proposed effects on metabolic pathways. Schematics show glycolysis and pyruvate metabolism annotated with the mRNAs and proteins analyzed in this study in cerebellum from 3- or 5-week old WT and *Npc1* ^-/-^ mice. Green squares: increased mRNA or protein levels. Red ovals: decreased mRNA or protein levels. No colour: unchanged mRNA or protein levels. PPP: pentose phosphate pathway. The grey shading represents the mitochondrial compartment. The proposed effects on the metabolic pathways are depicted by the thickness of the arrows.

Here we have investigated brain energy metabolism in a mouse model of NPC disease. Using an unbiased metabolomics strategy and targeted gene expression analyses of wildtype and *Npc1* ^-/-^ brain tissue, we found i) significant alterations in glucose and pyruvate metabolism that suggested a pre-symptomatic decrease in the oxidative metabolism of glucose, and a compensatory increase in glycolysis with disease progression, and ii) a pre-symptomatic activation of astrocytic antioxidant response systems, which suggests that oxidative stress plays a key role early in NPC disease pathogenesis. A graphic overview of the gene expression changes found in *Npc1* ^-/-^ cerebellum at 3 and 5 weeks of age is shown in Figure 8.

### Glycolysis and pyruvate oxidation in *Npc1^-/-^* cerebellum

The most striking alterations found in our metabolomics analysis were an increase in lactate and a decrease in acetate/acetyl-CoA levels in *Npc1* ^-/-^ cerebellum and cerebral cortex as early as 3 weeks of age (Fig 2). The decrease in acetate/acetyl-CoA pointed to an impaired oxidative decarboxylation of pyruvate to acetyl-CoA for further oxidation in the citric acid cycle. High lactate levels are often indicative of increased anaerobic glycolysis due to hypoxia or mitochondrial dysfunction, and have been observed in brain and cerebrospinal fluid in cerebral ischemia, during physiological aging, and in certain models of neurodegenerative diseases associated with mitochondrial dysfunction, such as Huntington disease [59]–[63]. In addition, lactate levels can increase transiently with increased neuronal activity [64]–[66].

The increased mRNA levels of *Ldha*, *Eno1*, and *Pfk* isoforms and the higher PFK1 protein levels observed in *Npc1* ^-/-^ cerebellum at 5 weeks of age indicated that glycolysis was upregulated and contributed to the higher lactate levels in *Npc1* ^-/-^ brain at this age (Fig 3 and 4). Similarly, published microarray data from *Npc1* ^-/-^ mouse liver show increased expression of several glycolytic enzymes, including *Pfk* and *Ldh* isoforms [67]. Given that *Ldha* and *Eno1* are predominantly expressed in glia, which are also the main glycolytic cell type and the main source of lactate in normal brain, the increased expression of these isoforms suggested an increase in glycolysis in *Npc1* ^-/-^ astrocytes. The increased 2-deoxyglucose uptake into primary *Npc1* ^-/-^ neurons (Fig 4D) suggested that glycolysis can also increase in neurons in *Npc1* ^-/-^ brain. Whereas in astrocytes, increased glycolysis may serve to provide lactate and other energy substrates to neurons [31], [68], [69], an upregulation of neuronal glycolysis can have detrimental effects by limiting the availability of glucose for the pentose phosphate pathway and thus for synthesis of NADPH and regeneration of glutathione [70].

Although lactate levels were increased at all stages of NPC disease (Fig 2), the upregulation of glycolysis in *Npc1* ^-/-^ cerebellum, at least on gene and protein expression level, appeared to occur during progression to the early symptomatic stage rather than pre-symptomatically. Thus, at 3 weeks of age, only *Eno1* mRNA levels were increased while *Pfkm* mRNA levels were even slightly decreased in *Npc1* ^-/-^ cerebellum (Fig 3). With progression from early symptomatic to late stage NPC disease, glycolytic gene expression in *Npc1* ^-/-^ cerebellum appeared to normalize, even though lactate levels remained elevated (Fig 3). A recent proteomics study using the same *Npc1* ^-/-^ mice found decreased levels of glycolytic PKM1/2 and PFK-M in *Npc1* ^-/-^ cerebellum at 1 and 3 weeks of age respectively [71], in agreement with our findings at 3 weeks. However, whereas our results suggest that glycolysis increases in the early stages of NPC disease, these authors concluded that glycolysis was generally downregulated in NPC disease, based on their findings of decreased levels of glycolytic triosephosphate isomerase in *Npc1* ^-/-^ cerebellum at 5 weeks of age, and of decreased deoxyglucose uptake in cultured primary fibroblasts from human NPC patients [71]. *In vivo* measurements of brain glucose uptake by 2-Deoxy-2-[^18^F]fluoro-d-glucose positron emission tomography have shown glucose hypometabolism in one patient with adult onset NPC disease [72], whereas two other case reports of NPC patients examined with the same technique found unaltered or increased glucose metabolism in the cerebellum and hypometabolism only in the cerebral cortex [73], [74]. Clearly, NPC1-deficiency alters glucose metabolism in a complex manner that is highly dependent on the stage of the disease and on the brain region examined. Further investigations by *in vivo* measurements of metabolic flux or by neuroimaging approaches are needed to clarify the role of glycolytic activity in the NPC1-deficient brain [75], [76].

We also observed an increase in the levels of the M2 isoform of pyruvate kinase (PKM2, Fig 4C) in *Npc1* ^-/-^ cerebellum. PKM2 is commonly expressed in embryonic and cancer tissue, and, in contrast to PKM1, it is under complex allosteric regulation to control the flux of glycolytic intermediates into biosynthetic pathways [77], [78]. The consequences of increased PKM2 levels in *Npc1* ^-/-^ cerebellum are unclear, but expression of an embryonic isozyme may point to disturbances in cell cycle or differentiation. Similarly, higher mRNA levels of the embryonic aldolase B have been found in *Npc1* ^-/-^ murine liver [67].

The increased lactate and decreased acetate/acetyl-CoA levels in *Npc1* ^-/-^ cerebellum at 3 weeks of age without a clear upregulation of glycolysis at this age (Fig 2 and 3) suggested that pyruvate metabolism was affected by NPC1-deficiency. Alterations in pyruvate metabolism were apparent in the markedly increased *Pcx* and decreased *Pdha*, *Me1* and *Me2* mRNA levels in *Npc1* ^-/-^ cerebellum at 3 weeks of age (Fig 5). The strong trend to increased inhibitory phosphorylation of PDH in *Npc1* ^-/-^ cerebellum at 3 weeks of age, the significantly increased phosphorylated PDH and the decreased total PDH protein in *Npc1* ^-/-^ cerebellum at 5 weeks of age (Fig 5) further indicated that an impaired oxidative decarboxylation of pyruvate to acetyl-CoA contributed to the high lactate and low acetate/acetyl-CoA levels in *Npc1* ^-/-^ cerebellum. Impaired PDH activity could also contribute to the decreased ATP generation in the presence of pyruvate/malate that we observed in assays with mitochondria isolated from *Npc1* ^-/-^ brain at 3 weeks of age (Fig 6). In a previous study, Cologna et al. also found significantly decreased PDH protein levels and a strong trend to increased pyruvate levels in the cerebella of 1-week old *Npc1* ^-/-^ mice [71]. Other reports have shown decreased mRNA expression of PDH isoforms in *Npc1* ^-/-^ cerebellum at a late stage of the disease, as well as in *Npc1* ^-/-^ liver [27], [67]. Moreover, increased mRNA levels of different PDH Kinase isoforms have been reported for liver samples from *Npc1* ^-/-^ mice aged 1 week and older, and for NPC1-deficient fibroblasts [67], [79]. Thus, a decline in PDH activity seems to be an early and persistent consequence of NPC1-deficiency in many cell types. Our finding that both *Pdk1* and *Pdk2* mRNA levels were increased in *Npc1* ^-/-^ cerebellum (Fig 5) suggested that PDH phosphorylation increased in both neurons and astrocytes, since neurons express PDK1, and PDK2 is predominantly present in astrocytes [54]. The upregulation of astrocytic *Pcx* in *Npc1* ^-/-^ cerebellum at 3 weeks of age and concomitant downregulation of *Me1* and *Me2* (Fig 5) may serve to counteract a build-up of pyruvate and the subsequent feedback inhibition of glycolysis, as has been described in other cell types [80].

The brain is highly sensitive to a decrease in PDH activity because of its strong reliance on the oxidative metabolism of glucose [28]. Congenital PDH deficiencies manifest with neurological symptoms such as ataxia and seizures [81], and decreases in PDH activity are also observed in Alzheimer disease and physiological aging [82], [83]. In astrocytes, PDH activity is normally kept low by high basal phosphorylation [54], and pyruvate is diverted into lactate formation and anaplerotic pyruvate carboxylation [84]. In neurons, however, maintenance of dephosphorylated, active PDH is essential to achieve the most energy-efficient complete oxidation of glucose [54]. Impaired oxidative decarboxylation of pyruvate also prevents the use of lactate or alanine as energy substrates, leaving increased glycolysis or oxidation of glutamine as the main alternative energy sources. Thus, a decrease in PDH activity could lead to impaired energy generation and/or to metabolic adaptations with possible consequences for neuronal redox status and neurotransmitter metabolism [85]. In addition, differences in neurotransmitter metabolism can also contribute to alterations in energy metabolism. Increased neuronal activity increases the neuronal energy requirement to maintain the membrane potential, and is commonly coupled to increased astrocytic lactate production [64], [65]. If the imbalance in neurotransmitter levels and in particular the decrease in inhibitory GABA that was observed in *Npc1* ^-/-^ cerebellum at 5 and 7 weeks of age (Fig 2) led to changes in neuronal activity, it could thus contribute to the increase in lactate production at later stages of the disease. On the other hand, in *Npc1* ^-/-^ hippocampus, GABA levels were decreased at 3 weeks of age without a concomitant increase in lactate (Figure S3). Moreover, a variety of homeostatic synaptic scaling mechanisms can stabilize overall neuronal firing through compensatory downregulation of excitatory activity [86]–[88].

Other factors could also contribute to impaired mitochondrial energy generation in *Npc1* ^-/-^ brain. Our findings of decreased *Mtco2* mRNA and increased *Ucp2* mRNA levels in *Npc1* ^-/-^ cerebellum at all ages (Fig 6) could indicate defects in cytochrome c oxidase/complex IV, and possibly increased uncoupling of the electron transport chain, even though the role of UCP2 in the brain is not quite clear. Cologna et al. found decreased ATP synthase levels in *Npc1* ^-/-^ cerebellum at 1 week of age, but no changes in ATP synthase levels at 3 weeks of age [71]. Using isolated brain mitochondria from 3-week old *Npc1* ^-/-^ mice, we found a decrease in ATP production with pyruvate and malate as energy substrates, but no differences in the presence of succinate. Even with the caveat that assays in isolated mitochondria are limited by their artificial environment, these findings suggest that impaired oxidative metabolism of pyruvate may be more prominent than electron transport chain deficiencies.

In several other neurodegenerative diseases associated with mitochondrial dysfunction, deficiencies in mitochondrial ATP production are associated with increased phosphorylation and activation of AMPK [89]–[93]. However, we observed no significant alterations in the levels of phosphorylated AMPK in cerebellum from 3-, 4- or 5-week old *Npc1* ^-/-^ mice (Fig 6), suggesting that any deficiencies in oxidative phosphorylation in *Npc1* ^-/-^ cerebellum were mild enough to be offset by compensatory metabolic adaptations. Such compensatory mechanisms could include the upregulation of *Pcx* in astrocytes in *Npc1* ^-/-^ cerebellum at 3 weeks of age and the upregulation of glycolytic gene expression (PFK, *Pfk, Ldha*) at 5 weeks of age (Fig 3 - 5), which could serve to counteract pyruvate build-up and energy deficits during the early symptomatic stage of NPC disease. Moreover, a proteomics analysis showed increased ATP synthase levels in *Npc1* ^-/-^ cerebellum at 5 weeks of age [71]. Our finding of increased mRNA levels of PGC-1α, a transcriptional coactivator for mitochondrial biogenesis, in *Npc1* ^-/-^ cerebellum at 5 weeks of age (Fig 6) suggested a compensatory upregulation of mitochondrial biogenesis, however, the lower levels of mitochondrial DNA observed in *Npc1* ^-/-^ cerebellum at the same age (Fig 6) indicated a decreased mitochondrial number or mitochondrial defects. A possible explanation for this seeming contradiction may come from recent work by Ordonez et al. showing an accumulation of depolarized, dysfunctional mitochondria due to defective mitophagy in NPC1-deficient human embryonic stem-cell derived neurons [24]. We speculate that with progression to late stage NPC disease, compensatory mechanisms begin to fail. Thus, at 7 weeks of age, PGC-1α mRNA levels in *Npc1* ^-/-^ cerebellum were decreased (Fig 6) and metabolic gene expression appeared mostly unaltered or decreased due to neuronal loss (Fig 3 and 5; *Eno2, Aralar1*). The lack of significant defects in ATP generation by mitochondria isolated from the brains of 7-week old *Npc1* ^-/-^ mice (this study, Fig 6), but decreased ATP generation by mitochondria isolated from brain homogenates of 9-week old *Npc1* ^-/-^ mice observed by Yu et al. [22], also indicate a progressive deterioration of mitochondrial quality.

### Early increase in oxidative stress and activation of antioxidant response systems

The most striking pre-symptomatic alterations in *Npc1* ^-/-^ cerebellum in mRNA and protein expression were related to oxidative stress and antioxidant response systems, for example increases in the levels of *Nfe2L2*, *Hmox1*, *Sod1*, and *Ucp2* mRNA and of pJNK and MnSOD protein (Fig 7). Increases in markers for oxidative stress have been found previously in several cell and animal models of NPC, as well as in human NPC patients [8], [25], [27], [67], [94], [95]. However, whereas our findings strongly suggest a role for oxidative stress early during NPC disease pathogenesis, a previous study by Cluzeau et al. had concluded that oxidative stress was a late occurrence in NPC disease, based on microarray data of liver taken from *Npc1* ^-/-^ mice between the ages of 1 and 11 weeks [67]. In that study, pathway maps related to oxidative stress showed significant alterations in *Npc1* ^-/-^ liver only at 11 weeks of age [67]. Even if the difference to our results may in part be due to methodological differences, the much later onset of oxidative stress in *Npc1* ^-/-^ liver compared to brain seems to suggest that in NPC disease, the cerebellum may be exposed to greater oxidative stress or be more sensitive to oxidative stress than the liver. Differences in the susceptibility to oxidative stress between mitochondria from liver and brain have been reported previously [96], [97]. A recent study investigated the effects of supplementing *Npc1* ^-/-^ mice with the antioxidant N-acetylcysteine beginning at 4 or 6 weeks of age, and noted a modest reduction in oxidative stress markers and a slight improvement in certain aspects of the NPC disease phenotype in the N-acetylcysteine-treated animals [98]. Our findings indicate that oxidative stress in the *Npc1* ^-/-^ cerebellum increases before the onset of overt neurological symptoms, and suggest that early antioxidant treatment may be beneficial in NPC disease.

Oxidative stress and mitochondrial dysfunction form a vicious cycle observed in many neurodegenerative disorders, where alterations in the electron transport chain and in oxidative phosphorylation lead to increased production of reactive oxygen species (ROS), which in turn promote further mitochondrial and metabolic dysfunction through oxidative modification and accelerated degradation of glycolytic and mitochondrial proteins [99]. Interestingly, PDH appears to be highly sensitive to oxidative modification, as seen following cerebral ischemia or in acutely respiring yeast [99]–[101]. JNK activation by oxidative stress can also decrease PDH activity. Thus, in cortical neurons, activated JNK translocates to the mitochondrial outer membrane and initiates a phosphorylation cascade that results in inhibitory PDH phosphorylation [83], [102]. In addition, JNK activation can increase PDK1 transcription [103].

The initial cause of a deterioration in mitochondrial quality in *Npc1* ^-/-^ brain is unclear. One possibility is that impaired mitophagy leads to the accumulation of dysfunctional mitochondria that produce more ROS [104]. Several groups, including ours, have also shown that cholesterol trafficking defects in NPC1-deficient cells can lead to an increase of cholesterol in mitochondria, which in turn may affect their function [20]–[23]. An upregulation of glycolysis in *Npc1* ^-/-^ neurons and/or decreased ME1 activity may further increase oxidative stress by decreasing NADPH synthesis for glutathione regeneration [70]. Initial increases in oxidative stress or initial metabolic alterations can thus cause further changes in energy metabolism and increase oxidative damage in *Npc1* ^-/-^ brain, leading to a cascade of subsequent adaptations as seen in many neurodegenerative diseases.

Our study also provides further evidence that both neurons and astrocytes are affected by NPC1-deficiency, which is in agreement with previous reports of early microglial and astrocyte activation [13], [57], [105], [106]. However, the observed pre-symptomatic metabolic alterations in astrocytes also raise questions as to the role of astrocytes in NPC pathology. On one hand, studies using chimeric mice [107], mice with cell-type specific deletion of NPC1 [108] or with cell-type specific expression of NPC1 in an NPC1-null background [109] have clearly demonstrated a neuron-inherent, glia-independent mechanism of Purkinje cell death in NPC1-deficient cerebellum. Moreover, depletion or expression of NPC1 in astrocytes alone neither caused nor rescued the NPC neurological phenotype [108], [109], with the exception of one report [110]. On the other hand, given the close interdependence of neurons and astrocytes, it seems unlikely that astrocytes could show such striking and early metabolic alterations, and yet have no impact on NPC disease progression. Our findings cannot resolve this quandary; however, they clearly show that many of the alterations in *Npc1* ^-/-^ astrocytes can be neuroprotective, including the activation of Nrf2 and upregulation of *Hmox1* (HO1) [111]–[114]. Nrf2 activity increases astrocytic synthesis and secretion of glutathione [111], [112], which, after hydrolysis, generates cysteine and glycine for neuronal glutathione synthesis [111], [112]. HO-1 also plays a mostly cytoprotective role, but it can also exacerbate neuronal and astrocytic injury after prolonged increased expression [115], [116], possibly contributing to the further deterioration seen at late stage NPC disease. For the development of additional therapeutic intervention strategies aimed at energy metabolism, it will be important to determine in more detail where and when different metabolic alterations develop, and whether these changes are potentially neuroprotective or part of the neuronal decline. Moreover, the significant alterations we observed prior to onset of symptoms once again underline the importance of early diagnosis and intervention.

## Supporting Information

Figure S1 **PPEDA scores plots for the ^1^H-NMR spectra of wildtype and** ***Npc1*** **^-/-^ cerebral cortex and hippocampus.** Scores plots representing the comparisons of wildtype (WT, blue squares) and *Npc1* ^-/-^ (red squares) samples at 3, 4, 5 and over 7 weeks of age as indicated. A) Cerebral Cortex samples. B) Hippocampal samples. Only sample sets of WT and *Npc1* ^-/-^ cerebral cortices taken at 7 weeks of age were statistically significantly different.
(TIF)

Figure S2 **Alterations in energy metabolite levels in** ***Npc1*** **^-/-^ cerebral cortex.** Aqueous extracts of the cerebral cortices from 3-, 4-, 5-, and 7-week old wildtype (WT) and *Npc1* ^-/-^ mice were analyzed by ^1^H-NMR spectroscopy. Spectra were deconvolved and integrated. Peak areas were standardized to total peak area. NAA: N-acetylaspartate. Inosit: myo-Inositol. Graphs in each column show data from the same set of WT and *Npc1* ^-/-^ mice of one age. The small numbers inside the bars for lactate indicate the number of mice in each group. Data are expressed as percent of the average of WT samples of the same age. Shown are the mean ± SEM. * p<0.05, *Npc1* ^-/-^ vs. WT.
(TIF)

Figure S3 **Alterations in energy metabolite levels in** ***Npc1*** **^-/-^ hippocampus.** Aqueous extracts of the hippocampi from 3- and 5-week old wildtype (WT) and *Npc1* ^-/-^ mice were analyzed by ^1^H-NMR spectroscopy. Spectra were deconvolved and integrated. Peak areas were standardized to total peak area. NAA: N-acetylaspartate. Inosit: myo-Inositol. Graphs in each column show data from the same set of WT and *Npc1* ^-/-^ mice of one age. The small numbers inside the bars for lactate indicate the number of mice in each group. Data are expressed as percent of the average of WT samples of the same age. Shown are the mean ± SEM. * p<0.05, *Npc1* ^-/-^ vs. WT.
(TIF)

Text S1 **Supporting methods.** Detailed description of the acquisition of ^1^H-NMR spectra and the statistical analysis by PPEDA.
(PDF)

Table S1 **Primer pairs used for qPCR analysis.** Listed are the sequences of the primer pairs and the annealing temperatures used for qPCR analysis as well as the accession numbers of the genes of interest.
(PDF)

Table S2 **Relative mRNA expression of potential housekeeping genes** ***Ppia*** **,** ***Actin*** **, and** ***Rpl13a*** **.** The mRNA levels of each of the three potential housekeeping genes *Ppia*, *Actin*, and *Rpl13a* were measured in wildtype (WT) and *Npc1* ^-/-^ cerebellum and their relative expression was calculated by the Pfaffl method with each of the other two as housekeeping genes.
(PDF)

Table S3 **Primary antibodies used in this study.** Listed are the sources for the primary antibodies used for immunoblotting.
(PDF)
