# Supplementary figures and images for: Pre-Symptomatic Activation of Antioxidant Responses and Alterations in Glucose and Pyruvate Metabolism in Niemann-Pick Type C1-Deficient Murine Brain
Source: PLoS One. 2013 Dec 18;8(12):e82685. doi: 10.1371/journal.pone.0082685 (PMC3867386; doi:10.1371/journal.pone.0082685)

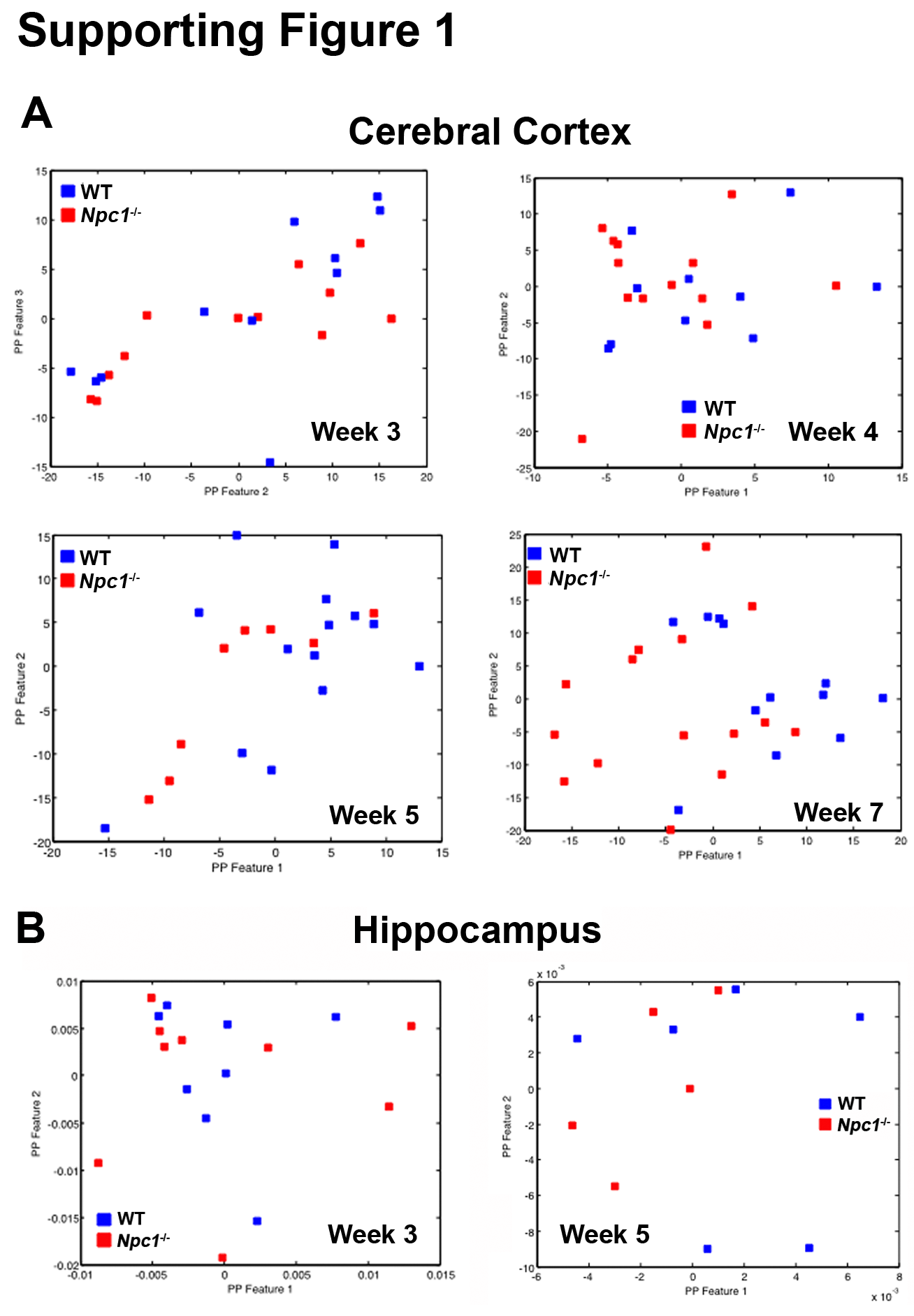

Supplement: Figure S1 — PPEDA scores plots for the 1H-NMR spectra of wildtype and Npc1 -/- cerebral cortex and hippocampus. Scores plots representing the comparisons of wildtype (WT, blue squares) and Npc1 -/- (red squares) samples at 3, 4, 5 and over 7 weeks of age as indicated. A) Cerebral Cortex samples. B) Hippocampal samples. Only sample sets of WT and Npc1 -/- cerebral cortices taken at 7 weeks of age were statistically significantly different. (TIF) [file pone.0082685.s001.tif]

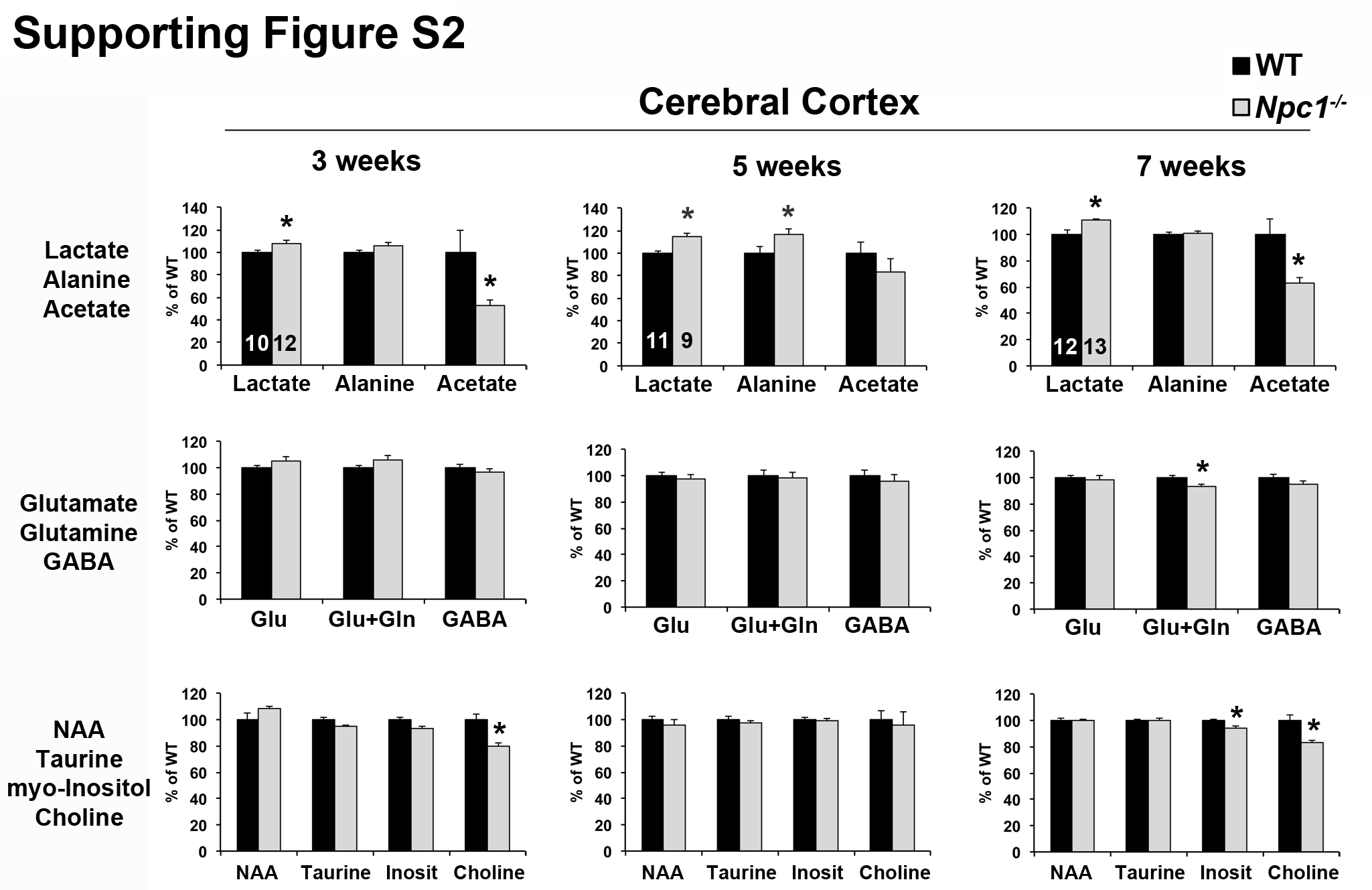

Supplement: Figure S2 — Alterations in energy metabolite levels in Npc1 -/- cerebral cortex. Aqueous extracts of the cerebral cortices from 3-, 4-, 5-, and 7-week old wildtype (WT) and Npc1 -/- mice were analyzed by 1H-NMR spectroscopy. Spectra were deconvolved and integrated. Peak areas were standardized to total peak area. NAA: N-acetylaspartate. Inosit: myo-Inositol. Graphs in each column show data from the same set of WT and Npc1 -/- mice of one age. The small numbers inside the bars for lactate indicate the number of mice in each group. Data are expressed as percent of the average of WT samples of the same age. Shown are the mean ± SEM. * p<0.05, Npc1 -/- vs. WT. (TIF) [file pone.0082685.s002.tif]

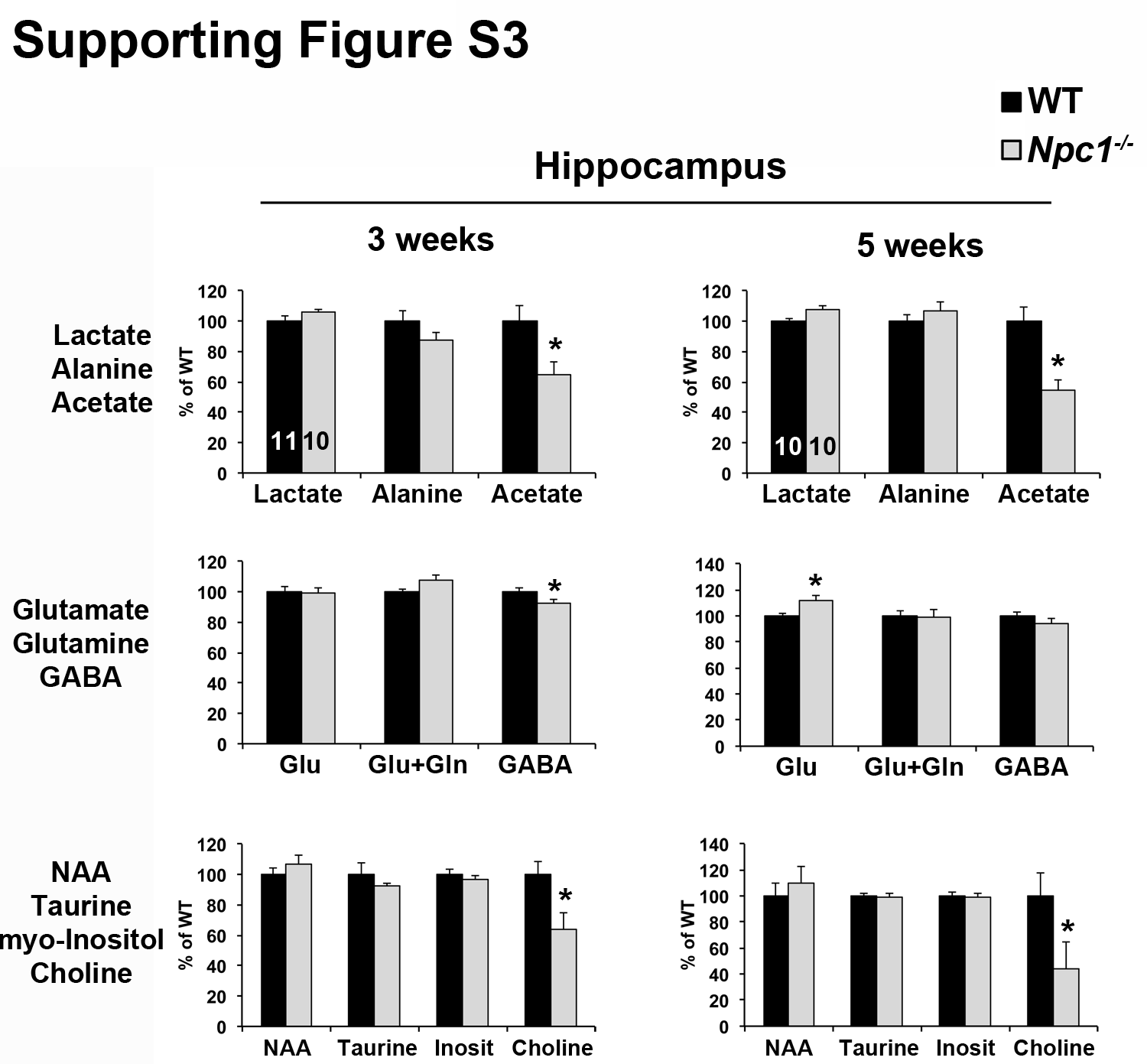

Supplement: Figure S3 — Alterations in energy metabolite levels in Npc1 -/- hippocampus. Aqueous extracts of the hippocampi from 3- and 5-week old wildtype (WT) and Npc1 -/- mice were analyzed by 1H-NMR spectroscopy. Spectra were deconvolved and integrated. Peak areas were standardized to total peak area. NAA: N-acetylaspartate. Inosit: myo-Inositol. Graphs in each column show data from the same set of WT and Npc1 -/- mice of one age. The small numbers inside the bars for lactate indicate the number of mice in each group. Data are expressed as percent of the average of WT samples of the same age. Shown are the mean ± SEM. * p<0.05, Npc1 -/- vs. WT. (TIF) [file pone.0082685.s003.tif]
