# Supplementary material for: Pre-Symptomatic Activation of Antioxidant Responses and Alterations in Glucose and Pyruvate Metabolism in Niemann-Pick Type C1-Deficient Murine Brain
Source: PLoS One. 2013 Dec 18;8(12):e82685. doi: 10.1371/journal.pone.0082685 (PMC3867386; doi:10.1371/journal.pone.0082685)
Supplement: Text S1 — Supporting methods. Detailed description of the acquisition of 1H-NMR spectra and the statistical analysis by PPEDA. (PDF) [file pone.0082685.s004.pdf]

Supporting Text S1

Supporting Methods:

Acquisition of ¹H-NMR spectra – Tissue samples a were transferred into FastPrep®-24 Lysis Matrix tubes (MP Biomedicals Inc., Solon, OH) for homogenization and mixed with 400 µL methanol and up to 125 µL water to a final methanol/water ratio of 3.2:1 (v/v). Samples were homogenized via ultra-sonication in the FastPrep®-24 instrument for 40 seconds at 6 m/s. Aqueous and organic phases were separated by extraction with 400 µL chloroform and 200 µL water. Aqueous extracts were dried under nitrogen, re-dissolved in 700 µL phosphate buffer (NaH_2PO_4, pH 7.2) with 172.2 mg/mL of sodium 3-trimethylsilyl-2,2,3,3-d_4-propionate (TMSP, Cambridge Isotope Laboratories, Andover, MA) in D_2O as internal reference standard, and stored in liquid nitrogen until analysis. 1D-^{1}H-NMR spectra were acquired using a 5 mm TCI CryoProbe™ (Bruker Biospin) with automatic tuning and matching, and a z-axis gradient amplifier and digital lock on a Bruker Avance III spectrometer (Bruker Biospin) operating at 700 MHz proton resonance frequency. Samples were added to 5 mm NMR sample tubes in a SampleJetTM sample changer (Bruker Biospin, Fällanden, Switzerland) pre-chilled SampleJetTM to 4°C. In automation, each sample was pre-warmed to 298.2 K for 5 min before acquisition of 1D-^{1}H-NMR spectra at 25°C using a 5 mm TCI CryoProbe™ (Bruker Biospin) with automatic tuning and matching and a z-axis gradient amplifier and digital lock on a Bruker Avance III spectrometer (Bruker Biospin) operating at 700 MHz proton resonance frequency. Tuning and matching to 50 Ω resistive impedance as well as shimming was applied under automation to each sample while the receiver gain was held constant for all samples. 1D ^{1}H-NMR spectra were acquired using a 12 µs (90°) pulse calibrated in automation using the pulsecal macro, 10 kHz spectral width and a 2 s relaxation delay with residual water pre-saturation (PS) using a CW irradiation attenuation of 60 dB during the relaxation delay, with 128 transients and 8 dummy scans collected into 32k data points. These were later zero-filled to 64k

and an exponential line-broadening of 0.3 Hz applied before Fourier transformation. Following manual zero- and first-order phasing, as well as manual baseline correction using TopSpinTM 2.1 (Bruker Biospin) the resulting spectra were calibrated (TMSP at δ = 0.0 ppm), also using TopSpinTM 2.1, before being exported to MATLAB 7.1 (MathWorks®, Natick, MA) using ProMetab 1.0 software26 for further processing.

Statistical analysis by principal component analysis (PCA) and projection pursuit for exploratory data analysis (PPEDA) – After manual phasing and baseline correction, the spectra were converted to an appropriate format for subsequent multivariate analyses using MATLAB® 7.1 software (MathWorks, Natick, MA) using an in-house written code. Each spectrum was segmented into 0.005-ppm chemical shift (bin size) between 0.200 and 10.000 ppm, and the spectral area within each bin integrated. Bins between 4.500–5.980 ppm containing the residual water resonances were removed. This localized each of the resonances that exhibit pH-induced chemical shift variability into single bins. Prior to principal component analysis (PCA), the data were pre-processed to identify outliers using Hotelling's T2 values, Q residuals [1] and plots of Studentized residuals vs. residual leverage [2]. Any spectrum that was flagged as an outlier was inspected to determine the cause for its outlying tendency and if found to be experimental, that sample was prepared again and a new spectrum acquired for it. Analysis of the data was carried out using PCA models constructed for the data consisting of an NMR spectrum for each sample extract. Each spectrum was normalized to unit sum and centered on the mean for the spectra of mouse brain tissue in each week. Scores plots for principal component (PC) and their respective loadings plots were generated and assessed for classification and metabolite identification. In addition, we analyzed the data using projection pursuit (PP) with a kurtosis projection index given that it is a more robust approach for exploratory analysis compared to PCA [3].

In general applications of PCA and PPEDA, PCs, organized as columns of the score matrix, are often plotted against each other to depict sample clusters. Alongside these, loadings are employed to depict the essence of the variable patterns, i.e., the relationship between the variable and sample spaces. Therefore the plots of loadings can be used to determine which peaks within, the NMR spectra, exhibit intensity changes that are correlated with the mutant vs. wildtype conditions. The plots represent a bio-molecular profile associated with $Npc1^{-/-}$ where peaks with positive or negative loadings indicate their significance to the groups that are identified in the respective quadrants within the PC plots.

Statistical significance of these metabolite intensities was further determined by comparison of a select metabolites from $Npc1^{-/-}$ and age-matched wildtype control using two-tailed, T-test with a p-value set at the 95% CI and corrected for multiple comparisons using the Bonferroni correction. The p-value was set at p = 0.05/m, where m is the number of hypotheses tested.

REFERENCES

(1) Macho, S., F. Sales, M. Callao, M. Larrechi, and F. Rius. (2001) Outlier Detection in the Ethylene Content Determination in Propylene Copolymer by Near-Infrared Spectroscopy and Multivariate Calibration. Applied Spectroscopy 55: 1532-1536.

(2) Beebe, K., R. Pell, and M. Seasholtz. 1998. Chemometrics: A Practical Guide. John Wiley & Sons.

(3) Daszykowski, M., B. Walczak, and D. Massart. (2003) Projection Methods in Chemistry. Chemometrics and Intelligent Laboratory Systems 65: 97-112.
