# Supplementary material for: Pre-Symptomatic Activation of Antioxidant Responses and Alterations in Glucose and Pyruvate Metabolism in Niemann-Pick Type C1-Deficient Murine Brain
Source: PLoS One. 2013 Dec 18;8(12):e82685. doi: 10.1371/journal.pone.0082685 (PMC3867386; doi:10.1371/journal.pone.0082685)
Supplement: Table S1 — Primer pairs used for qPCR analysis. Listed are the sequences of the primer pairs and the annealing temperatures used for qPCR analysis as well as the accession numbers of the genes of interest. (PDF) [file pone.0082685.s005.pdf]

Supporting Table S1: List of primers used for SYBR-Green qPCR analysis

Target	Gene	Forward Primer (5' → 3')	Reverse Primer (3' → 5')	T_{Anneal} (^{o}C)	Accession Number
α-α-Enolase	*Eno1*	GTACATCACTCCCGACCAGC	CGCAGCCTTGGCAATC	58.6	NM_023119
Aralar	*Slc25a12*	TGCAGCTCGGGTGTTTC	AAGTCTGTAGCCACCGATGTG	56.3	NM_172436
β-Actin	*Actb*	AGCCTTCCTTCTTGGGTATGG	ACACAGAGTACTTGCGCTCAG	54.6	NM_007393
Cytochrome C Oxidase (Subunit 2)	*Cox2*	GAGTCGTTCTGCCAATAGA	ATGGGCATAAAGCCTATGGT	52.7	AF378830
Cyclophilin	*Ppia*	TCTTCTTGCTGGTCTTGCCATTCC	TCCAAAGACAGCAGAAAACTTTCG	55.0	NM_008907
Glial Fibrillary Acidic Protein	*Gfap*	TGCTGGAGGGCGAAGAAA	CGGATCTGGAGGTTGGAGAA	54.0	NM_001131020
Glucose-6-Phosphate Dehydrogenase	*G6pd2*	AGCCTCCTACAAGCACCTCA	TGGTTCGACAGTTCATTGGA	55.7	NM_019468
Heme Oxygenase 1	*Hmox1*	GGCGTCACTTCGTCAGAG	ACTGGAGGAGCGGTGTC	57.1	NM_010442
Hexokinase 1	*Hxk1*	TGGGCACCATGATGACTTGTG	TCGTCCAGACAGCCGTTGTC	57.2	NM_001146100+010438
Hypoxia-Induced Factor 1α	*Hif1a*	CCTGCGTGCATGTCTAATC	ATGTGCCATGTACCAGAATC	53.2	NM_01031
Lactate Dehydrogenase	*Ldha*	TGGGAGAACATGGCGACTC	GCACCCGCCTAAGGTTCTTC	58.7	NM_010699
Malic Enzyme (Cytosolic)	*Me1*	ATTCGAGGCGTTTCGTTG	CTGTTGCGTTACTGGTTGAC	52.9	NM_008615
Malic Enzyme (Mitochondrial)	*Me2*	AAGGCGCTGACGACTCA	CGGCAGCAGGGAAACGTAG	55.3	NM_145494
Malic Enzyme (Mitochondrial, neuronal)	*Me3*	CATGAGCGCCCTATCGTC	GATCCCACCAGCGATGAC	57.9	NM_181407
Mitochondrial Genome	*Mtco1*	TGCTAGCCGAGGCATTAC	GGGTGCCCAAAGAATCAGAAC	60.0	NC_005089
NADH Dehydrogenase Flavoprotein	*Ndufv1*	CTTCCCCACTGGCCTCAAG	CCAAAACCCAGTGATCCAGC	60.0	NM_133666
Neurofilament Heavy Polypeptide	*Nefh*	CAGACATTGCCTCCTACCAG	GGCCATCTTGACGTTGAG	55.2	NM_010904
Neuronal Enolase	*Eno2*	AAGCCATCCAAGCGTGCAAGC	CGCCAGTCTTGATCTGGCCTGT	60.0	NM_013509
NRF2	*Nrf2*	TTGGCAGGAGCTATTTTCC	GAACAGCGGTAGTATCAGC	50.1	NM_010902
PGC1α	*Ppargc1a*	ATTGAGAGACCGCTTTGAAG	TCGACCTGCGTAAAGTATATCC	53.4	NM_008904
Phosphofructokinase 1	*Pfkl*	TTGACTGCAGGACCAATG	CGGTAGACATCACGTAGCTTC	55.4	NM_008826
Phosphofructokinase 1	*Pfkm*	TAAGATGGGTGCTAAGGCTATG	GATCAATCTCGTACTTGGCTAGG	55.3	NM_001163487
Phosphofructokinase 1	*Pfkp*	AGCACCGTGTCCATTCGATAG	CTTCAGCTCTGCCACTGGTTG	55.0	NM_019703
Pyruvate Carboxylase	*Pcx*	TGGACGTGGCAGTAGACTC	CATGGTAGCCGTGCAATC	57.7	NM_001162946
Pyruvate Dehydrogenase	*Pdha*	CTGGCATAAACCCTACGGAC	CGCCTTTCCCTTTAGCAC	56.4	NM_008810
Pyruvate Dehydrogenase Kinase 1	*Pdk1*	ATACACTGCCAATGATTGAC	GCCATGCCGCTGTAAC	53.1	NM_172665
Pyruvate Dehydrogenase Kinase 2	*Pdk2*	TACCTCAGCCGCATCTC	TGGCGTTGGTGGCATTGAC	55.0	NM_133667

Target	Gene	Forward Primer (5’ → 3’)	Reverse Primer (3’ → 5’)	T_{Anneal} (^{o}C)	Accession Number
Pyruvate Kinase Type M	*Pkm*	TGCCGTGACTCGAAATCCC	GGCCAAGTTTACACGAAGGTC	55.0	NM_001253883+011099
RPL13a	*Rpl13a*	AGGTTGTTCGGCTGAAGC	TTCTTCTTCCGATAGTGCATC	54.9	NM_009438
Superoxide Dismutase 1	*Sod1*	ATGAAAGCGGTGTGCGTG	TGCTGGCCTTCAGTTAATCC	56.4	NM_011434
Uncoupling Protein 2	*Ucp2*	TCCACGCAGCCTCTACAATG	TGGAAGCGGACCTTTACCAC	60.3	NM_011671
