# Supplementary material for: Pre-Symptomatic Activation of Antioxidant Responses and Alterations in Glucose and Pyruvate Metabolism in Niemann-Pick Type C1-Deficient Murine Brain
Source: PLoS One. 2013 Dec 18;8(12):e82685. doi: 10.1371/journal.pone.0082685 (PMC3867386; doi:10.1371/journal.pone.0082685)
Supplement: Table S2 — Relative mRNA expression of potential housekeeping genes Ppia , Actin , and Rpl13a . The mRNA levels of each of the three potential housekeeping genes Ppia, Actin, and Rpl13a were measured in wildtype (WT) and Npc1 -/- cerebellum and their relative expression was calculated by the Pfaffl method with each of the other two as housekeeping genes. (PDF) [file pone.0082685.s006.pdf]

Supporting Table S2: Relative expression of different housekeeping genes

**Target: cyclophilin (*Ppia*)**	**Per *Actin***	**Per *Rpl13a***
3 week *Npc1*$^{-/-}$ vs WT	1.0 ± 0.14	**0.8 ± 0.15***
5 week *Npc1*$^{-/-}$ vs WT	0.9 ± 0.08	1.1 ± 0.12
7 week *Npc1*$^{-/-}$ vs WT	0.9 ± 0.09	1.0 ± 0.12

**Target: Actin (*Actin*)**	**Per *Ppia***	**Per *Rpl13a***
3 week *Npc1*$^{-/-}$ vs WT	1.0 ± 0.07	**0.8 ± 0.06**
5 week *Npc1*$^{-/-}$ vs WT	1.1 ± 0.10	1.3 ± 0.18
7 week *Npc1*$^{-/-}$ vs WT	1.0 ± 0.14	1.0 ± 0.23

**Target: RPL13A (*Rpl13a*)**	**Per *Ppia***	**Per *Actin***
3 week *Npc1*$^{-/-}$ vs WT	**1.4 ± 0.17**	**1.5 ± 0.18**
5 week *Npc1*$^{-/-}$ vs WT	1.2 ± 0.15	0.9 ± 0.11
7 week *Npc1*$^{-/-}$ vs WT	1.1 ± 0.15	1.0 ± 0.10

Supporting Table S2: To test whether the different housekeeping genes were differentially expressed in wildtype (WT) and *Npc1*$^{-/-}$ cerebellum, we calculated the expression of each housekeeping gene measured (*Ppia*, *Actin*, and *Rpl13a*) in *Npc1*$^{-/-}$ cerebellum per WT of the same age using the Pfaffl method with each of the other two as housekeeping gene. *Rpl13a* appeared markedly increased in the cerebella from 3-week old *Npc1*$^{-/-}$ mice compared to WT (WT samples = 1.0), and was therefore unsuitable as a housekeeping gene.
