# Supplementary material for: Pre-Symptomatic Activation of Antioxidant Responses and Alterations in Glucose and Pyruvate Metabolism in Niemann-Pick Type C1-Deficient Murine Brain
Source: PLoS One. 2013 Dec 18;8(12):e82685. doi: 10.1371/journal.pone.0082685 (PMC3867386; doi:10.1371/journal.pone.0082685)
Supplement: Table S3 — Primary antibodies used in this study. Listed are the sources for the primary antibodies used for immunoblotting. (PDF) [file pone.0082685.s007.pdf]

Supporting Table S3: Antibody sources

Target	Source
Actin	Santa Cruz Biotechnology, sc1616
AMPKα	Cell Signaling #2603
phospho-AMPKα (Thr172)	Cell Signaling #2535
AMPKβ1/2	Cell Signaling #4150
phospho-AMPKβ (Ser108)	Cell Signaling #4181
Hexokinase 1	Santa Cruz, sc46695
MnSOD	Novus Biologicals NB100-1992
Phosphofructokinase PFKP	Santa Cruz Biotechnology sc130227
Pyruvate dehydrogenase E1α	Cell Signaling #2784
phospho-Pyruvate dehydrogenase E1α (Ser232)	Calbiochem AP1063
Pyruvate Kinase M1/2	Cell Signaling #3190
Pyruvate Kinase M2	Cell Signaling #3198
SAPK/JNK	Cell Signaling #9252
phospho-SAPK/JNK (Thr183/Tyr185)	Cell Signaling #9255
VDAC1	Abcam ab15895

Supporting Table S3: Antibodies used in this study.
